# Optimal dose and type of exercise improve the overall balance in adults with Parkinson’s disease: a systematic review and Bayesian network meta-analysis

**DOI:** 10.1007/s10072-025-08244-1

**Published:** 2025-05-27

**Authors:** Xianjin Fan, Yuan Yuan, Ying Bai, Chen Cheng, Junyu Wang, Tao Wang, Yong Yang, Ko-Chia Chen

**Affiliations:** 1 School of Physical Education and Sport, Chaohu University, No. 1 Xuefu Road, Chaohu Economic Development Zone, Hefei, 238000 Anhui Province China; 2 Department of Physical Education, Kunsan National University, Kunsan, 541150 Korea; 3 School of Tennis, Wuhan Sports University, Wuhan, 430079 China; 4 School of Exercise and Health, Shanghai University of Sport, Shanghai, 200438 China; 5 College of Physical Education and Health, Southwest University of Science and Technology, Mianyang, 621010 China; 6 Department of Sport, Leisure and Health Management, Tainan University of Technology, No.529, Zhongzheng Rd., Yongkang District, Tainan City, 710302 Taiwan (R.O.C.)

**Keywords:** Parkinson's disease, Exercise, Balance test batteries, Dose–response, RCTs, Bayesian network meta-analysis

## Abstract

**Objective:**

To investigate the dose–response relationship between various exercise modalities and their effectiveness in enhancing balance in Parkinson's disease (PD) patients.

**Methods:**

Searches were conducted in PubMed, Medline, Embase, PsycINFO, Cochrane Library, and Web of Science from inception to July 23th, 2024. Data were analyzed using R software, with the MBNMA and RJAGS packages to compute standardized mean differences (SMD) and 95% credible intervals (95% CrI). The network's risk of bias was rigorously evaluated by two independent reviewers using the ROB2 tool.

**Results:**

The analysis included 73 studies with 3,747 PD patients, assessing the effects of different exercises on balance. The findings demonstrate a nonlinear increasing dose–response relationship. Dance was the most effective, with an optimal dose of 1000 METs-min/week, closely followed by aerobic exercise (AE) at 1200 METs-min/week. Aquatic exercise (AQE), multicomponent exercise (MulC), and sensory exercise (SE) exhibited an inverted U-shaped response, with peak improvements at 700 METs-min/week for AQE, 610 METs-min/week for MulC, and 620 METs-min/week for SE. Balance and gait training (BGT), mind–body exercise (MBE), and resistance training (RT) also significantly improved balance, with optimal dosages below 750 METs-min/week.

**Conclusion:**

Specific exercises, particularly dance at 1000 METs-min/week, significantly enhance balance in PD patients. Nonlinear dose–response patterns and inverted U-shaped curves were evident, supporting the necessity for personalized exercise prescriptions based on the minimal clinically important difference (MCID) identified for AE, AQE, MBE, MulC, and SE, to optimize therapeutic outcomes.

**PROSPERO registration number:** CRD42024517241.

**Supplementary Information:**

The online version contains supplementary material available at 10.1007/s10072-025-08244-1.

## Introduction

Balance entails the task of keeping the body's center of gravity aligned within the support base boundaries during sitting or standing, as well as managing the center of gravity when transitioning to a new support base during activities like walking or running [1]. Everyday activities like walking or standing from a chair involve managing one's center of gravity as one transitions to a new base of support. Assessing an individual's ability to perform these tasks can serve as an indirect measure of their balance, especially for those with mobility impairments. However, balance difficulty is prevalent among patients diagnosed with Parkinson’s disease (PD), especially as the disease progresses into its middle and advanced stages [2]. As neurons degenerate and dopamine levels continue to decline [3], balance deteriorates as well. The decline in balance is closely linked with an increased risk of falls, diminished mobility, disability, and a decrease in overall quality of life among individuals with PD [4]. Therefore, balance in PD should receive more attention as a basis for treatment [5].

Exercise had emerged as a promising adjunctive therapy, demonstrating effectiveness in enhancing balance and mobility among individuals with PD [6]. However, different exercises seem to have different effects on balance improvement in PD. Experimental studies had demonstrated that aerobic and treadmill training can significantly improve balance in PD [7, 8], and a meta-analysis of Zhen et.al. results proved also that aerobic exercise was effective in improving patients'balance (including 20 original studies) [9]. Even though several studies had confirmed the efficacy of aerobic exercise on balance in PD, but the head-to-head RCT study of Christian et.al. found that resistance exercise was seen to be more effective than aerobic in improving balance in PD [10]. Additionally, a study by Lisa et.al. showed mind–body exercises like Yoga, Taichi, and qigong to improve balance in PD patients offer updates on clinical trials [11]. Meanwhile, dance, as a diverse intervention, was also shown to be effective in improving balance in PD in a pooled analysis of 372 PD patients [12]. Interestingly, a systematic review and meta-analysis by Lucia et. al. found a slight to moderate benefit of aquatic exercise over land exercise in improving balance [13]. Surprisingly, multicomponent exercises and sensory exercises with their intended unique benefits are becoming more and more popular in addressing balance issues in PD, and the network meta-analysis of Y.J. Qian et. al. found multicomponent exercise as the most effective intervention to improve Balance in PD (including 199 original studies) [14], Not only that, the study of Yong Y et. al. also gave good references through sensory exercise in terms of improving PD balance [15].

Different exercises have different effects on improving balance in PD, which may be due to different exercise dosages in different forms of exercise. In previous dose–response meta-analyses, most of them use time, frequency and period as the main results to explore dose–response [16, 17], or use meta-regression to find whether dose is a potential factor affecting the outcome [18]. None of these can subjectively reflect exercise dose–response relationships. However, the dose–response network meta-analysis of Daniel et.al. two studies, which provided a new direction for solving the exercise dose–response relationship [19, 20], for the first time, exercise dose was classified into task metabolic equivalents (METs) to explore the dose–response relationship, which standardized unit of energy consumption for the human body. It quantifies the intensity of different physical activities based on the resting metabolic rate [21]. Compared with exercise dosage based on a single dimension such as exercise frequency (times/week), single duration (minutes) or total number of intervention weeks, MET comprehensively considers the intensity, frequency and duration of exercise, and can more comprehensively reflect the total intensity of exercise received by individuals during the intervention process [22]. In person with Parkinson’s disease, little was known about how exercise-specific dosage interventions can improve balance. Therefore, in order to accurately determine the optimal exercise dose to increase balance in PD, the exercise dose was precisely calculated as the metabolic equivalents of the intervention task (number of interventions per week × minutes × METs of different forms of exercise = METs-min/week), the frequency, intensity, duration and type of exercise (such as aerobic and resistance training) may have different degrees of impact on the balance ability of PD patients, which has been confirmed by the study of Yuan et.al [23]. The general concept of"exercise is beneficial"is transformed into an actionable and precise treatment plan. Traditional PD management relies on drugs, and exercise intervention often remains at the level of vague suggestions (such as"moderate exercise every week"), lacking personalized guidance for different patient stages and symptom characteristics. Dose–response studies can establish a stratified intervention strategy by analyzing the association between exercise type, intensity, frequency and clinical outcomes (such as fall rate and balance score).

In order to fill this gap, in this systematic review and network meta-analysis, we employ new methodologies, including model-based dose–response network meta-analysis within a Bayesian framework [24], to explore the connection between different doses of exercise interventions and balance tests in PD. This study statistically significant improvements in balance do not always equate to clinical relevance. Therefore, our analysis emphasizes clinical importance in addition to statistical outcomes. Specifically, we aimed to identify the minimum clinically important difference (MCID) for balance measures and to determine the most effective exercise regimens for achieving clinically meaningful improvements. These findings may help patients and clinicians judge whether an intervention yields practically meaningful benefits and support evidence-based exercise recommendations for managing motor symptoms in PD.

## Method

This systematic review and network meta-analysis were prospectively registered with the International Prospective Register of Systematic Reviews (PROSPERO) (registration number: CRD42024517241). The network meta-analysis (NMA) adhered to the reporting guidelines outlined by the Preferred Reporting Items for Systematic Review and Meta-analysis Protocols statement extension for the PRISMA-NMA checklist [25].

### Search strategy

We systematically searched PubMed, Medline, Embase, PsycINFO, Cochrane Central Register of Controlled Trials, and Web of Science from inception to July 23 th, 2024. We have provided a complete search strategy (different databases, search dates, entries, and processes) in Supplementary File 1. Two investigators (Xianjin Fan and Ying Bai) independently screened the title/abstract/full text in duplicate, with any disagreements resolved by discussion or adjudication by a third author (Ko-Chia Chen).

### Selection criteria

We included studies based on the following criteria: (1) Participants: the study population consisted of patients diagnosed with Parkinson's disease (PD), with a mean age of ≥ 60 years, a Hoehn and Yahr stage < 4, and follow up conditions; (2) Intervention: any form of exercise was utilized as an intervention (comprising 8 distinct exercise types, detailed in Supplementary File 2); (3) Comparator: the comparator group did not receive the intervention and could involve usual care, health education, or active control (involving either a different type of exercise from the experimental group or the same type of exercise but with a different exercise dose); (4) Outcome: studies must report on at least one indicator from balance test batteries, including"Mini-BESTest, BESTest, BBS, Tinetti assessment scale, Fullerton Advanced Balance, Tinetti-gait and balance tests"; (5) Study design: only randomized controlled trials (RCTs) were considered.

Exclusion criteria were applied as follows: (1) Patients with PD who had concurrent other diseases (for instance, metabolic disease, cardiovascular diseases, stroke, Alzheimer’s disease, dementia); (2) Studies incorporating mixed interventions from different disciplines (e.g., combining exercise with repetitive transcranial magnetic stimulation); (3) Studies with exercise doses lacking explicit description of the type of exercise or inability to compute; (4) Non-RCTs.

### Data collection

In the process of data collection, two authors independently collected data from the included studies (Y.Y. and J.Y.W.). And all authors resolved disagreements by consensus. From each included study, we extracted the study (first author and year of publication), sample (sample size), age (patient age), intervention characteristics (invention duration/time, type of exercise), and outcome indicators. All detailed basic information was collated using standardized tables in Excel and included studies are provided in Supplementary File 3. Secondly, the amount of baseline and post-training change between the experimental group and control group were calculated by the following formula:

$$\begin{array}{c}{\text{Mean}}_{\text{change}}={\text{Mean}}_{\text{posttraining}}-{\text{Mean}}_{\text{baseline}}\\ {\text{SD}}_{\text{change}}=\sqrt{{\text{SD}}_{\text{baseline}}^{2}+{\text{SD}}_{\text{posttraining}}^{2}-2*\text{R}*{\text{SD}}_{\text{baseline}}*{\text{SD}}_{\text{posttraining}}}\end{array}$$

where R is a constant (R = 0.5) [26]. To meet the data analysis requirements of the Dose–Response Network meta-analysis package in the R program, we also converted the standard errors (SE), $SE=\frac{SD}{\sqrt{Sample size}}$[27]. When it was not possible to retrieve the minimum required data from published reports for dose–response meta-analyses, we contacted the authors and invited them to provide additional data.

### Data setting and management

The interventions were set according to the specific type of exercise performed:"Aerobic exercise (AE)","Aquatic exercise (AQE)","Balance and gait training (BGT)","Resistance Training (RT)","Dance","Mixed (Mul C, combining 2 or more specific types of movement)","Sensory Exercise (SE)","Mind Body Exercise (MBE: including"Qigong","Tai Chi", and"Yoga"). Then, different exercise interventions were categorized by specific types and doses, expressed in METs-min/week (metabolic equivalents of task). MET is a unit used to quantify energy expenditure during physical activity. 1 MET corresponds to the resting metabolic rate, approximately 3.5 mL O₂/kg/min or 1 kcal/kg/h. The weekly exercise dose (in METs-min/week) was calculated based on the product of session duration, frequency, and intensity for each exercise type [22, 28, 29]. To reduce heterogeneity in intervention characteristics and improve the connectivity of the network—both of which are essential for the validity of network meta-analysis—the estimated METs-min/week values were approximated to standardized dose levels. We used approximate values of 250, 500, 750, 1000, or 1500 METs-min/week, consistent with previous studies [19, 30].

### Data synthesis

We completed the data analysis for this study in R software (R Foundation for Statistical Computing, Vienna, Austria), and we employed “MBNMA” package and “rjags” package in R for conducting a Bayesian-Model of network meta-analysis [31, 32]. We compared fit indices for different models in common and random effects (Emax, Exponential, Restricted cubic spline, Non-parametric, and Linear models), including the Deviance Information Criterion (DIC), between-study standard deviation, number of parameters in the model, and residual values, as suggested by [33], we provided assessment results in the Supplementary File 5. Ultimately, we opted for the random effects model for the restricted cubic spline to evaluate the non-linear dose–response association. At the same time, we checked the connectivity of the motion pattern grid for different motion doses based on the dose approximation [34]. We assessed the two models by comparing them with an uncorrelated mean-effects model (UME) [35]. Finally, we assessed transitivity using a node-splitting approach [36]. There are no parametric results that indicate a violation of the above key assumptions and we did a detailed explanation of the statistical principles in different assumptions (Supplementary File 4).

In order to estimate the different exercise doses that resulted in the predicted maximum significant effect referred to as the ‘optimal exercise dose’ on balance test batteries in PD. We summarized the result of the dose–response relationship by the MCMC (Markov Chain Monte Carlo iterations) model (3 chains, 20000 iterations each (first 10000 discarded), n. thin = 10) of the beta coefficients on the restricted cubic spline curves [37].We positioned the three nodes at the 10 th, 50 th, and 90 th percentile of the exercise dose to visualize our results. Additionally, the effect sizes measure chooses the standardized mean difference (SMD) of the change score (end end-point minus baseline score) because the studies use different rating scales or units of the outcome.

To further enhance the clinical relevance of our findings, we conducted an estimation of the exercise dosage or range of dosages required to achieve the MCID, as recommended by JA Bernstein and DT Mauger [38]. In our analysis, we applied a distribution-based method to establish a consolidated MCID value for the overall balance, following the approach outlined by JA Watt, AA Veroniki, AC Tricco and SE Straus [39]. Our results indicated that the MCID for the Berg Balance Scale could be estimated as an improvement of 2.9 when considering a 0.4 standard deviation (SD) threshold, or an improvement of 3.6 at a 0.5 SD level (${\text{SD}}_{\text{pooled}}=\sqrt{\sum \left({n}_{i}-1\right)S{D}_{i}^{2}/ \sum \left({n}_{i}-1\right)}$). However, in order to provide clinicians with more robust and clinically meaningful guidance, we ultimately selected an MCID of 3.6 at the 0.5 SD threshold. Then, we calculated the pooled effect size (SMD) of the studies that at least achieved the estimated pooled MCID (k = 14). Finally, we predicted at which dose(s) of treatment these effects were achieved for each type of intervention [40, 41].

### Risk of bias and quality of evidence

The risk of bias was assessed according to the second version of the Cochrane risk-of-bias tool for randomized trials (ROB2) [42]. Two reviewers (Y.B. and Y.Y) assessed the study. The assessment items included randomized sequence generation, bias due to deviation from the intended intervention, incomplete data, bias in measurements, and selective bias in reporting results. Disagreements were resolved by a third author (J.Y.W.).

## Results

### Characteristics of included studies

**Fig. 1:**
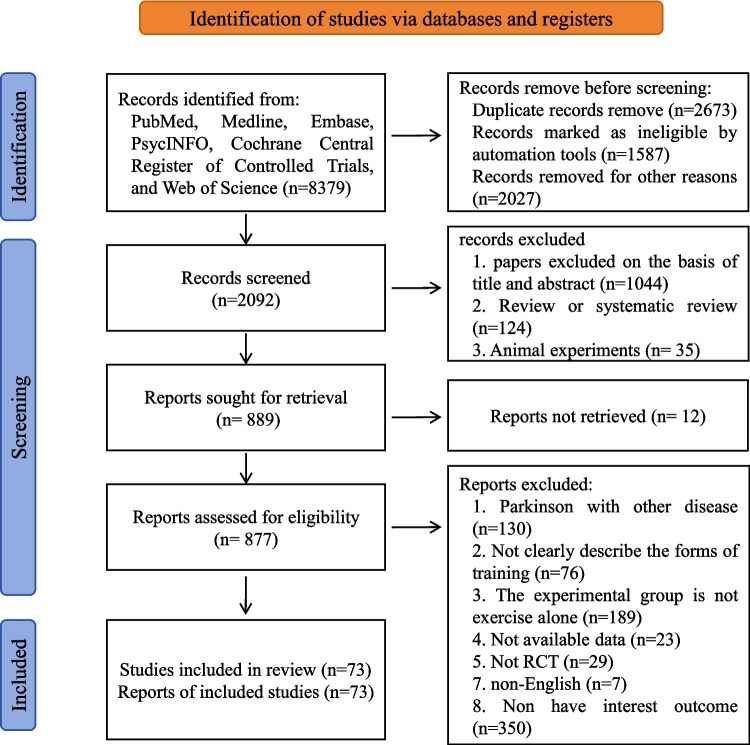
PRISMA Flow diagram of the search process for studies. *RCT* randomized controlled trials

A total of 8379 articles were retrieved from the databases. After removing duplicates, 877 articles were screened by title, abstract, and full text. After removing studies that did not meet the inclusion criteria, finally, 73 studies were included in our analysis. We provided an included studies list in Supplementary File 3**.** Involve 3747 PD patients were included in our studies. All patients included in the studies had PD and were aged between 57 to 78.3 years old with a mean disease duration of 8.4 years, and a mean of 2.4 for Hoehn and Yahr stages. The flow diagram of the search process for the systematic review study is presented in Fig. 1. We provided a basic characteristic of all included population studies in Supplementary File 3.

### Network connectivity

**Fig. 2:**
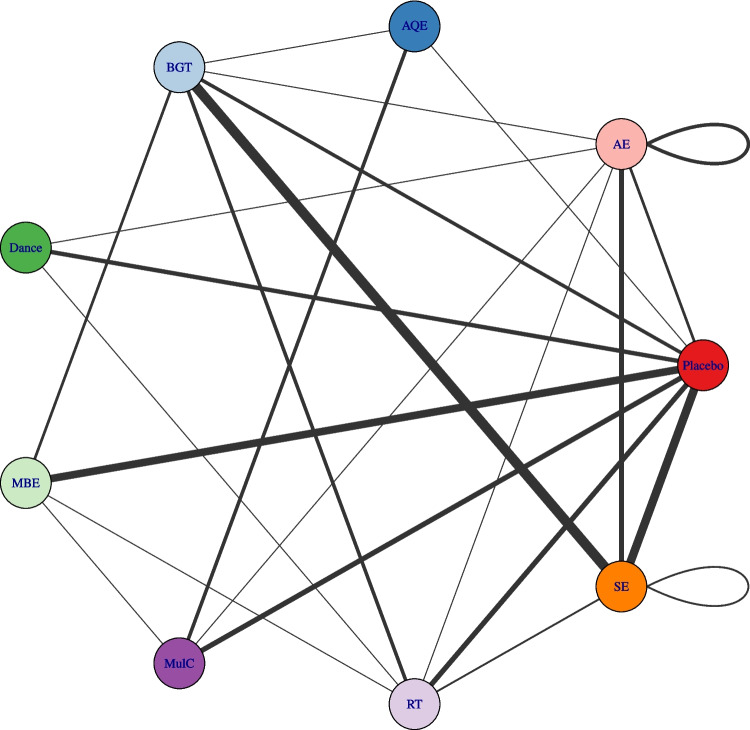
Agent-level network plot. AE Aerobic Exercise, AQE Aquatic Exercise, BGT Balance and Gait Training, Dance, MBE Mind–body Exercise, MulC Multicomponent Exercise Program, RT Resistance Training, SE Sensory Exercise, CON Control group. The thickness of the line represents the number of studies that included the intervention. Different colors may indicate differences in categories (such as different types of training methods)

**Fig. 3:**
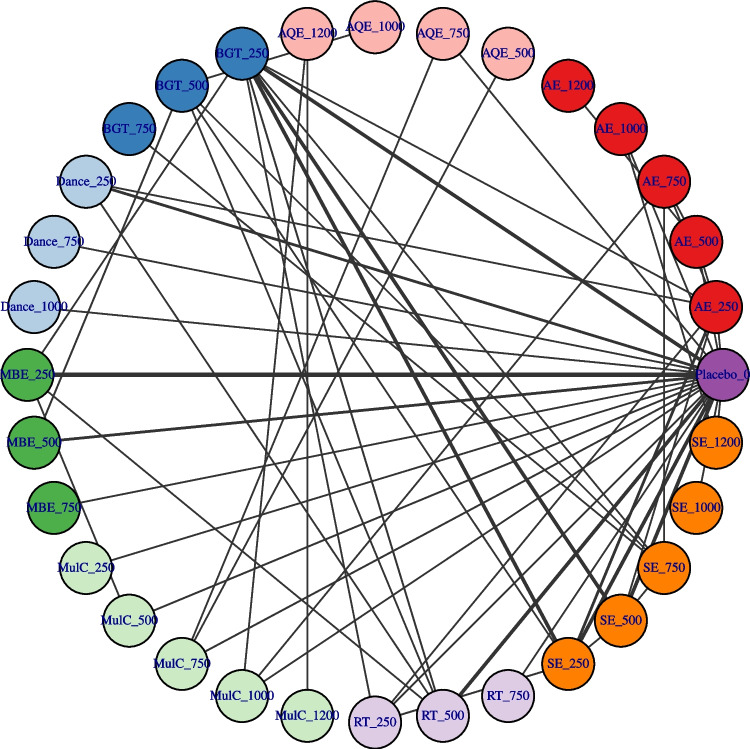
Treatment-level network plot. The first value indicates the specific intervention and the second one is the corresponding dose of that intervention. AE Aerobic Exercise, AQE Aquatic Exercise, BGT Balance and Gait Training, Dance, MBE Mind–body Exercise, MulC Multicomponent Exercise Program, RT Resistance Training, SE Sensory Exercise, CON Control group. The thickness of the line represents the number of studies that included the intervention. Different colors may indicate differences exercise and dose in categories (such as different types of training methods)

In dose response network meta-analysis, the core role of MET-min/week as an approximation is to address the heterogeneity of exercise interventions, and the assessment of network connectivity is a key step to ensure the reliability of the results, and this also step directly impact on the transitivity, consistency and dose response model fitting [43]. At the same time, whether or not connectivity is met determines the basis of the step in network meta-analysis (NMA) [36]. When direct comparison is not possible, a lack of connectivity can lead to low statistical power and misleading results. The results showed that there was no connectivity deficit in the two networks, thus ensuring the accuracy of the analysis (Fig. 2 and Fig. 3). In addition, this has been well confirmed in previous studies [19, 44].

### Dose–response relationship

**Fig. 4:**
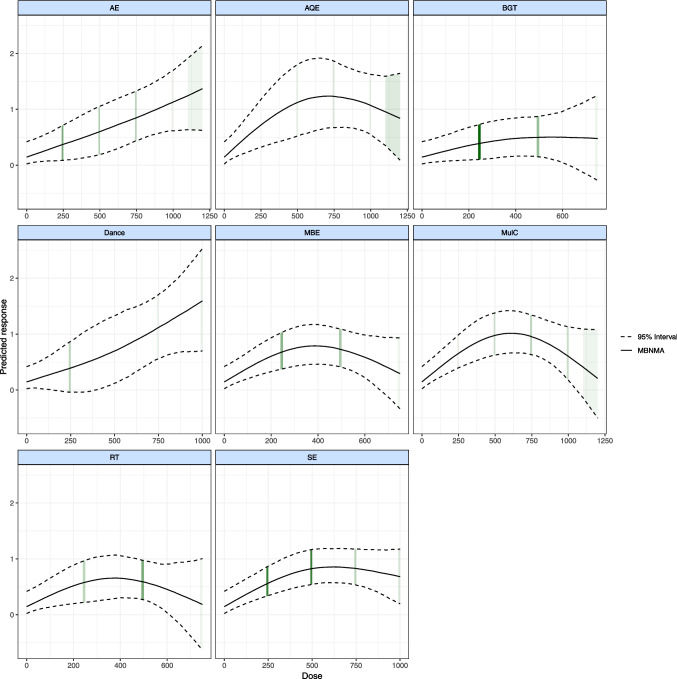
Dose–response association between treatment-level dose and change in the overall balance in Parkinson's disease patients

Figure 4 shows the nonlinear dose–response relationship between various exercise modalities and improvements in overall balance, with the intensity of green shading indicating the sample size for exercise doses—darker shades represent larger sample sizes. Analysis revealed that the doses for AE, AQE, Dance, MulC, and SE exceed 1000 METs-min/week, highlighting a nonlinear relationship between exercise dose and balance enhancement. Specifically, in 6 studies involving 121 patients with PD who participated in Dance, the effective dose range was estimated to be 470 to 1000 METs-min/week, with the optimal dose identified at 1000 METs-min/week (SMD: 1.632, 95% Crl: 0.663 to 2.619). Similarly, in 13 studies involving 314 patients with PD who participated in AE, the effective dose range was up to 1200 METs-min/week (SMD: 1.362, 95%Crl: 0.574 to 2.188), which was also the optimal dose for enhancing overall balance in PD.

Conversely, AQE, MulC, and SE displayed an inverted U-shaped response, indicating an optimal dose range for balance improvement. In 6 studies with 90 PD participants, AQE was found to effectively improve balance at any tested dose, with an optimal dose at 700 METs-min/week (SMD: 1.265, 95%Crl: 0.605 to 1.947). In 12 studies involving 510 PD patients, MulC showed an effective dose range up to 1000 METs-min/week, with the optimal dose at 610 METs-min/week (SMD: 1.025, 95%Crl: 0.615 to 1.461). Furthermore, in 26 studies with 694 PD participants, SE had an effective dose range up to 1000 METs-min/week, with the optimal dose at 620 METs-min/week (SMD: 0.862, 95%Crl: 0.573 to 1.182).

Additionally, BGT, MBE, and RT demonstrated effectiveness at lower doses. Specifically, in 21 studies involving 343 PD patients, BGT was effective up to 590 METs-min/week (SMD: 0.479, 95%Crl: 0.016 to 0.983), which was also identified as the optimal dose. It is worth attention that MBE and RT exhibited an inverted U-shaped dose–response, with optimal doses at 390 METs-min/week (SMD: 0.795, 95%Crl: 0.438 to 1.181) for MBE and 380 METs-min/week (SMD: 0.639, 95%Crl: 0.247 to 1.054) for RT in 12 studies each, involving 246 and 194 PD patients respectively.

### Treatment dose and minimal clinically important difference

The pooled effect size equivalent to the estimated MCID for total balance was moderate (k = 14, SMD = 0.66; 95% CrI [0.51 to 0.82]). Specifically, the MCID for Dance was identified at doses exceeding 470 METs-min/week. Furthermore, AE demonstrated a significant clinical effect on balance improvement at doses beyond 550 METs-min/week. In the case of AQE, the MCID was observed at doses exceeding 210 METs-min/week. For MBE, the MCID was noted at doses from 230 to 550 METs-min/week. The MCID for MulC ranged from 250 to 1000 METs-min/week, while SE showed an MCID at doses exceeding 310 METs-min/week. Notably, BGT and RT did not exhibit an MCID at any investigated dose.

### Risk of bias and quality of evidence

**Fig. 5:**
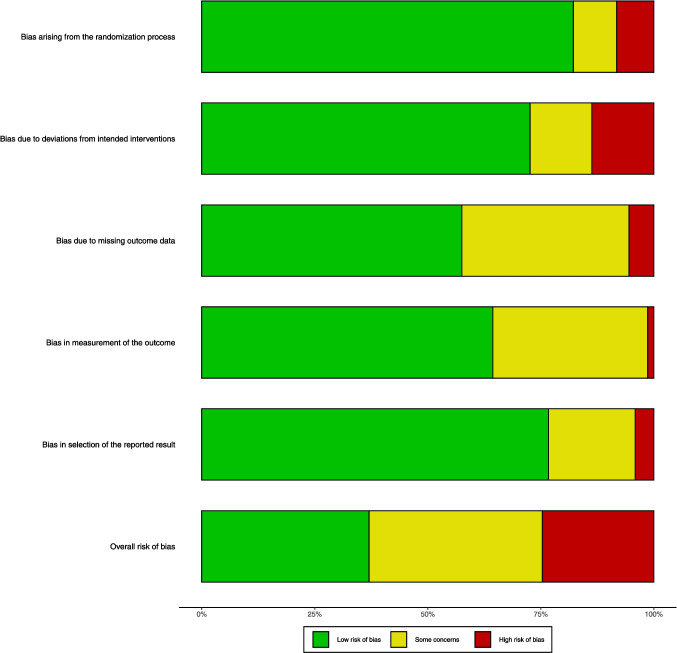
Cochrane risk of tool

Overall, 28 studies (37%) were classified a low risk of bias,26 studies (37%) were classified unclear risk of bias, and 19 studies (25%) were classified high risk of bias, Fig. 5 shows the result of Cochrane Risk of Bias Tool and Study-level risk of bias assessments are presented in **Supplementary File 6.**

## Discussion

This study has unearthed several critical insights that significantly advance our understanding of exercise interventions in improving balance for patients with PD. Firstly, our comprehensive analysis substantiates that all eight types of exercise incorporated in this study markedly enhance balance, solidifying the role of physical activity as a beneficial therapeutic approach for this population. Notably, dance emerges as the foremost intervention, with the optimal engagement quantified at 1000 METs-min/week. Secondly, the dose–response relationships for five types of exercises—AQE, MBE, MulC, RT, and SE—display an inverted U-shaped curve. This delineates a critical dosage threshold beyond which additional exercise yields no further balance improvements, highlighting the necessity for tailored exercise prescriptions based on patient-specific optimal dosage ranges. Thirdly, the establishment of the MCID for balance improvements is a significant stride towards contextualizing the clinical impacts of exercise. Our findings reveal that six exercise modalities (Dance, Aerobic Exercise, AQE, MBE, MulC, and SE) surpass this clinically important threshold, providing a robust basis for clinicians and therapists to formulate precise, evidence-based treatment plans. Collectively, these findings not only corroborate the positive impacts of exercise on motor symptoms in PD but also refine our recommendations regarding exercise types and their respective dosages to attain clinically meaningful outcomes, thereby aiming to elevate the quality of life for individuals afflicted with this condition.

One of the most pivotal outcomes of this study is the identification of the optimal types of exercise and their dose–response relationships for improving balance in patients with PD. Our findings underscore the significant advantages of specific exercises, notably AE and dance, in enhancing balance capabilities, as preliminarily identified in our prior network meta-analyses [45]. The results presented here not only reinforce these earlier findings but also delineate a more detailed, nonlinear dose–response pattern observed with AE and dance. Specifically, dance emerges as the superior modality for ameliorating balance in PD patients, demonstrating efficacy within a range of 470 to 1000 METs-min/week, with the optimal dose pinpointed at 1000 METs-min/week. Dance, a form of aerobic exercise, inherently incorporates rhythmic aerobic activity [46] which has been shown to significantly improve motor coordination and neurological function in PD [47–49]. The neuroprotective effects of aerobic exercises stem from increased neuroplasticity, enhanced mitochondrial function, and elevated levels of brain-derived neurotrophic factor (BDNF) [50], which are crucial for neuronal health and the maintenance of motor pathways affected by PD [51]. Moreover, dance adds an element of rhythmic and motor control to traditional aerobic exercise, which requires precise movements and coordination. This not only aids in the strengthening of neuromuscular control but also enhances proprioception and the integration of sensory feedback, vital for maintaining and improving postural stability in PD patients [52, 53]. These additional components of dance could explain its heightened efficacy, positioning it as a potentially superior intervention for improving balance in this population. The multifaceted demands of dance, involving coordination, rhythm, and motor control, extend its benefits beyond those of conventional aerobic activities, making it an integral part of therapeutic strategies aimed at ameliorating the motor symptoms of PD.

Interestingly, our study found that AQE, MBE, MulC, RT, and SE all exhibit an inverted U-shaped dose–response relationship in enhancing balance abilities in PD patients. This phenomenon suggests there is an optimal exercise dosage, beyond which no additional benefits are derived and may indeed lead to decreased gains or increased risks of fatigue and injury. The commonality among these exercises that results in the inverted U-shaped curve likely originates from their demands on both the physiological and neurological systems. Each modality focuses on complex, controlled movements that challenge the neuromuscular and cognitive systems concurrently [54]. This dual demand can lead to enhanced motor control and neuroplasticity up to a certain point, after which the stress on the neuromuscular system might outweigh the neuroprotective and neuroenhancing benefits [55]. This aligns with existing research on exercise physiology and neuroplasticity in PD, which suggests that moderate, regular stimulation of neural pathways can delay degeneration and improve function, while overstimulation may deplete neuronal reserves [51, 56]. The inverted U-shaped dose–response curve emphasizes the importance of precision in prescribing exercise regimens. Although exercise can improve balance and overall health in PD patients, excessive intensity or duration might trigger stress responses that are counterproductive. By identifying the optimal dose range, therapists can maximize the benefits of exercise while minimizing risks, thereby ensuring sustainable and effective intervention strategies for managing and potentially ameliorating PD symptoms.

This understanding is pivotal in translating the theoretical insights from our dose–response findings into practical clinical applications. As we transition into the implications of these findings, it is essential to highlight that defining the MCID for balance improvements further aids in fine-tuning exercise recommendations to achieve clinically meaningful outcomes, ensuring that each patient's treatment plan is as effective and tailored as possible [57]. Our study has established the MCID for balance improvements in PD patients, revealing that while all exercise modalities statistically enhanced balance, only dance, AE, AQE, MBE, MulC, and SE achieved the MCID. This differentiation highlights the clinical relevance of these exercises in producing not just statistically significant, but also practically significant improvements that can profoundly affect patients'quality of life. Thus, the application of MCID in clinical guidelines involves integrating these findings into patient care strategies, facilitating the development of targeted exercise programs that not only meet statistical thresholds for effectiveness but also reach meaningful clinical thresholds that translate into better patient outcomes [58, 59]. This approach ensures that therapeutic exercises are not only scientifically valid but also tailored to meet the specific clinical needs and improvement markers that are most relevant to the patient’s condition and lifestyle.

This study presented several noteworthy strengths, we utilized advanced Bayesian network meta-analysis techniques, we conducted a detailed comparison of the effects of different types of exercises on the balance abilities of PD patients, providing valuable insights into their relative efficacy and optimal dosages. The adoption of stringent inclusion criteria and rigorous methodologies has enhanced the reliability and validity of our study findings. However, this study is not without its limitations. Firstly, although we endeavored to define the METs for each exercise based on the activity and type, METs values are calculated based on average data. These values may not accurately reflect individual differences due to age, gender, weight, fitness level, health status, and disease progression, which can influence METs discrepancies. In addition, due to the limited number of included articles, the exercise doses of RT, BGT, and MBE were all relatively low, with an upper limit of 750 METs-min/week. Therefore, the effects of higher doses of RT, BGT, and MBE on balance ability in PD patients remain unclear, and further RCT intervention studies are necessary to determine their effects. Future studies should build on the results of this study to determine personalized exercise programs for PD patients with different personal characteristics and motor symptoms. It is crucial to tailor exercise interventions to maximize balance ability and improve the quality of life of PD patients in the next step.

## Conclusion

In this systematic review and network meta-analysis, we carefully evaluated various exercise interventions aimed at improving balance in PD patients, including 73 studies with 3747 participants. Our findings identify dance as the most effective exercise modality for enhancing balance in PD patients, showing peak efficacy at a dosage of 1000 METs-min/week. Furthermore, we established the MCID for balance improvements, highlighting the clinical significance of customizing exercise prescriptions to achieve meaningful patient outcomes. These insights enable clinicians and therapists to develop precise, evidence-based exercise programs to alleviate balance issues in PD patients, thus enhancing their quality of life and reducing the risk of falls.

## Supplementary Information

Below is the link to the electronic supplementary material.

Supplementary file1 (DOCX 275 KB)

## Data Availability

This published article and its supplementary information include all data generated or analyzed during this study.

## Abbreviations

RCT: Randomized controlled trials
SMD: Standardized mean difference
SD: Standardized Deviation
SE: Standard errors
AE: Aerobic exercise
AQE: Aquatic Exercise
BGT: Balance and Gait Training
CON: Control group
MBE: Mind-body exercise
MulC: Mixed training
RT: Resistance training
SE: Sensory exercise
DIC: Deviance information criterion
UME: Uncorrelated mean-effects model
MCMC: Markov Chain Monte Carlo
MCID: Minimal clinically important difference
METs: Metabolic equivalent of task
PD: Parkinson’s disease
