# Supplementary material for: Optimal dose and type of exercise improve the overall balance in adults with Parkinson’s disease: a systematic review and Bayesian network meta-analysis
Source: Neurol Sci. 2025 May 27;46(9):4169–80. doi: 10.1007/s10072-025-08244-1 (PMC12394369; doi:10.1007/s10072-025-08244-1)
Supplement: Supplementary file 1 — Supplementary file1 (DOCX 275 KB) [file 10072_2025_8244_MOESM1_ESM.docx]

**Optimal dose and type of exercise improve overall balance in adults with Parkinson’s disease: a systematic review and Bayesian network meta-analysis**

[Supplementary File 1: Search Strategy 2](#_Toc185063121)

[1.1 Database: PubMed <inception to July 23 2024> 2](#_Toc185063122)

[1.2 Database: Ovid MEDLINE(R) <1946 to July 23 2024> 5](#_Toc185063123)

[1.3 Database: Embase <1974 to 7](#_Toc185063124)

[1.4 Database: PsycINFO <1806 to July 16 2024> 9](#_Toc185063125)

[1.5 Cochrane 11](#_Toc185063126)

[1.6 Database: Web of Science <1965 to July 20 2024> 12](#_Toc185063127)

[Supplementary File 2: Definitions of exercise types and non-exercise training control 14](#_Toc185063128)

[Supplementary File 3: Table 1: Characteristics of included studies 16](#_Toc185063129)

[Table 2: Inclusion of study-specific data 26](#_Toc185063130)

[List of included studies 37](#_Toc185063131)

[Supplementary File 4: Three key assumptions of network meta-analysis in our study 43](#_Toc185063132)

[(1) Consistency Test 43](#_Toc185063133)

[Table 4.1. Consistent and UME models fit the comparison 43](#_Toc185063134)

[Figure 4.1: Validation Model Consistency Scatterplot 43](file:////Users/demon/Desktop/张志扬/投稿稿件/投稿/20241030/PeerJ/Supplementary%20File.docx#_Toc185063135)

[(2) Transitivity Test 44](#_Toc185063136)

[Table 4.2: Node-splitting analysis of inconsistency 44](#_Toc185063137)

[Figure 4.2: Node splitting analysis (density plot) 49](file:////Users/demon/Desktop/张志扬/投稿稿件/投稿/20241030/PeerJ/Supplementary%20File.docx#_Toc185063138)

[Supplementary 5: The relationship of Non-linear model 50](#_Toc185063139)

[Figure 5.1: “Split” NMA of different exercise treatment agents 50](#_Toc185063140)

[Table 5.1: the fit indices from each of the models fitted 51](#_Toc185063141)

[Figure 5.2: Deviance box plot at different exercise levels 52](#_Toc185063142)

[Supplementary File 6: Study-level risk of bias analysis 53](#_Toc185063143)

[Supplementary File 7: PRISMA Checklist 55](#_Toc185063144)

# **Supplementary File 1: Search Strategy**

## 1.1 Database: PubMed <inception to July 23 2024>

***Search Strategy:***

| #25 | Search: ((Parkinson disease[MeSH Terms]) AND ((((((((((exercise*[MeSH Terms]) OR (resistance training[MeSH Terms])) OR (Tai Ji[MeSH Terms])) OR (Qigong[MeSH Terms])) OR (Exercise Movement Techniques[MeSH Terms])) OR (Yoga[MeSH Terms])) OR (Virtual Reality[MeSH Terms])) OR (hydrotherapy[MeSH Terms])) OR (Dance Therapy[MeSH Terms])) OR ("aerobic exercise" or "aquatic exercise" or "balance training" or "body weight support treadmill" or "gait **training" or "high-speed resistance training" or "multicomponent exercise program" or "multidisciplinary exercise program" or "Nordic Walking" or Physiotherapy or pilates or "power training" or "Robotic-assisted gait training" or stretch or Tango or "treadmill training" or "walking" or "whole body vibration"))) AND ((((((((randomized controlled trial[**Publication Type]) OR (controlled clinical trial[Publication Type])) OR (randomized[Title/Abstract])) OR (placebo[Title/Abstract])) OR (randomly[Title/Abstract])) OR (trial[Title])) OR (clinical trials as topic[MeSH Terms])) NOT ((animals[MeSH Terms]) NOT (humans[MeSH Terms]))) | 890 |
| --- | --- | --- |
| #24 | Search: (((((((randomized controlled trial[Publication Type]) OR (controlled clinical trial[Publication Type])) OR (randomized[Title/Abstract])) OR (placebo[Title/Abstract])) OR (randomly[Title/Abstract])) OR (trial[Title])) OR (clinical trials as topic[MeSH Terms])) NOT ((animals[MeSH Terms]) NOT (humans[MeSH Terms])) | 1,341,164 |
| #23 | Search: ((((((randomized controlled trial[Publication Type]) OR (controlled clinical trial[Publication Type])) OR (randomized[Title/Abstract])) OR (placebo[Title/Abstract])) OR (randomly[Title/Abstract])) OR (trial[Title])) OR (clinical trials as topic[MeSH Terms]) | 1,449,600 |
| #22 | Search: (((((((((exercise*[MeSH Terms]) OR (resistance training[MeSH Terms])) OR (Tai Ji[MeSH Terms])) OR (Qigong[MeSH Terms])) OR (Exercise Movement Techniques[MeSH Terms])) OR (Yoga[MeSH Terms])) OR (Virtual Reality[MeSH Terms])) OR (hydrotherapy[MeSH Terms])) OR (Dance Therapy[MeSH Terms])) OR ("aerobic exercise" or "aquatic exercise" or "balance training" or "body weight support treadmill" or "gait training" or "high-speed resistance training" or "multicomponent exercise program" or "multidisciplinary exercise program" or "Nordic Walking" or Physiotherapy or pilates or "power training" or "Robotic-assisted gait training" or stretch or Tango or "treadmill training" or "walking" or "whole body vibration") | 551,542 |
| #21 | Search: (animals[MeSH Terms]) NOT (humans[MeSH Terms]) | 4,815,925 |
| #20 | Search: humans[MeSH Terms] | 19,183,084 |
| #19 | Search: animals[MeSH Terms] | 23,999,009 |
| #18 | Search: clinical trials as topic[MeSH Terms] | 355,600 |
| #17 | Search: trial[Title] | 238,308 |
| #16 | Search: randomly[Title/Abstract] | 356,459 |
| #15 | Search: placebo[Title/Abstract] | 223,336 |
| #14 | Search: randomized[Title/Abstract] | 561,707 |
| #13 | Search: controlled clinical trial[Publication Type] | 617,986 |
| #12 | Search: randomized controlled trial[Publication Type] | 528,725 |
| #11 | Search: "aerobic exercise" or "aquatic exercise" or "balance training" or "body weight support treadmill" or "gait training" or "high-speed resistance training" or "multicomponent exercise program" or "multidisciplinary exercise program" or "Nordic Walking" or Physiotherapy or pilates or "power training" or "Robotic-assisted gait training" or stretch or Tango or "treadmill training" or "walking" or "whole body vibration" | 361,398 |
| #10 | Search: Dance Therapy[MeSH Terms] | 396 |
| #9 | Search: hydrotherapy[MeSH Terms] | 20,257 |
| #8 | Search: Virtual Reality[MeSH Terms] | 2,684 |
| #7 | Search: Yoga[MeSH Terms] | 3,002 |
| #6 | Search: Exercise Movement Techniques[MeSH Terms] | 8,700 |
| #5 | Search: Qigong[MeSH Terms] | 229 |
| #4 | Search: Tai Ji[MeSH Terms] | 1,183 |
| #3 | Search: resistance training[MeSH Terms] | 9,538 |
| #2 | Search: exercise*[MeSH Terms] | 297,336 |
| #1 | Search: Parkinson disease[MeSH Terms] | 69,308 |

## 1.2 Database: Ovid MEDLINE(R) <1946 to July 23 2024>

***Search Strategy: --------------------------------------------------------------------------------***

1 Parkinson$.mp. (136173)

2 exp parkinson disease/ (69312)

3 (aerobic exercise or aquatic exercise or balance training or body weight support treadmill or Dance Therapy or exercise$ or Exercise Movement Techniques or gait training or high-speed resistance training or hydrotherapy or multicomponent exercise program or multidisciplinary exercise program or Nordic Walking or Physiotherapy or pilates or power training or Qigong or resistance training or Robotic-assisted gait training or stretch or tai ji or Tango or treadmill training or walking or Virtual Reality or whole body vibration or Yoga).mp. (540012)

4 exp resistance training/ (9532)

5 exp exercise$/ (206975)

6 exp tai ji/ (1182)

7 exp Qigong/ (228)

8 exp Exercise Movement Techniques/ (8695)

9 exp Yoga/ (2999)

10 exp Virtual Reality/ (2682)

11 exp hydrotherapy/ (20254)

12 exp Dance Therapy/ (396)

13 randomized controlled trial.pt. (527440)

14 controlled clinical trial.pt. (94123)

15 randomized.ab. (517037)

16 clinical trials as topic.sh. (195553)

17 randomly.ab. (355668)

18 trial.ti. (238446)

19 exp clinical trial/ (888782)

20 exp randomized controlled trials/ (145969)

21 exp cross-over studies/ (49955)

22 (clinic$ adj2 trial).mp. (746815)

23 (random$ adj5 control$ adj5 trial$).mp. (770827)

24 (crossover or cross-over).mp. (100433)

25 randomi$.mp. (943880)

26 (random$ adj5 (assign$ or allocat$ or assort$ or reciev$)).mp. (256419)

27 1 or 2 (136173)

28 3 or 4 or 5 or 6 or 7 or 8 or 9 or 10 or 11 or 12 (586531)

29 13 or 14 or 15 or 16 or 17 or 18 or 19 or 20 or 21 or 22 or 23 or 24 or 25 or 26 (1770322)

30 27 and 28 and 29 (1246)

1.3 Database: Embase <1974 to July 18 2024>
***Search Strategy:***

--------------------------------------------------------------------------------

1 Parkinson$.mp. (219918)

2 exp parkinson disease/ (163492)

3 (aerobic exercise or aquatic exercise or balance training or body weight support treadmill or Dance Therapy or exercise$ or Exercise Movement Techniques or gait training or high-speed resistance training or hydrotherapy or multicomponent exercise program or multidisciplinary exercise program or Nordic Walking or Physiotherapy or pilates or power training or Qigong or resistance training or Robotic-assisted gait training or stretch or tai ji or Tango or treadmill training or walking or Virtual Reality or whole body vibration or Yoga).mp. (816955)

4 exp resistance training/ (20137)

5 exp exercise$/ (363106)

6 exp tai ji/ (3173)

7 exp Qigong/ (836)

8 exp Exercise Movement Technique/ (82933)

9 exp Yoga/ (8492)

10 exp Virtual Reality/ (18896)

11 exp hydrotherapy/ (3829)

12 exp Dance Therapy/ (527)

13 randomized.ab. (757440)

14 randomly.ab. (481077)

15 trial.ti. (332106)

16 exp clinical trial/ (1627821)

17 exp randomized controlled trials/ (200725)

18 exp cross-over studies/ (66963)

19 (clinic$ adj2 trial).mp. (1639410)

20 (random$ adj5 control$ adj5 trial$).mp. (961796)

21 (crossover or cross-over).mp. (123532)

22 randomi$.mp. (1323771)

23 (random$ adj5 (assign$ or allocat$ or assort$ or reciev$)).mp. (209949)

24 1 or 2 (219918)

25 3 or 4 or 5 or 6 or 7 or 8 or 9 or 10 or 11 or 12 (841979)

26 13 or 14 or 15 or 16 or 17 or 18 or 19 or 20 or 21 or 22 or 23 (2779253)

27 24 and 25 and 26 (2562)

## 1.4 Database: PsycINFO <1806 to July 16 2024>

***Search Strategy:***

| Set No. Searched for Databases Results | | | |
| --- | --- | --- | --- |
| S1 | Parkinson* | APA PsycInfo® | 39453 |
| S2 | mainsubject(parkinson disease) | APA PsycInfo® | 25842 |
| S3 | su((aerobic exercise or aquatic exercise or balance training or body weight support treadmill or Dance Therapy or exercise$ or Exercise Movement Techniques or gait training or high-speed resistance training or hydrotherapy or multicomponent exercise program or multidisciplinary exercise program or Nordic Walking or Physiotherapy or pilates or power training or Qigong or resistance training or Robotic-assisted gait training or stretch or tai ji or Tango or treadmill training or walking or Virtual Reality or whole body vibration or Yoga)) | APA PsycInfo® | 63328 |
| S4 | su(exercise$) | APA PsycInfo® | 39377 |
| S6 | su(physical activity) | APA PsycInfo® | 39490 |
| S7 | ab(randomized) | APA PsycInfo® | 83500 |
| S8 | ab(randomly) | APA PsycInfo® | 75844 |
| S9 | ti(trial) | APA PsycInfo® | 41193 |
| S10 | ab(clinical trial) | APA PsycInfo® | 51602 |
| S11 | ab(randomized controlled trials) | APA PsycInfo® | 36991 |
| S12 | ab(cross-over studies) | APA PsycInfo® | 2076 |
| S13 | ab(crossover studies) | APA PsycInfo® | 5363 |
| S14 | ab(randomi*) | APA PsycInfo® | 83917 |
| S15 | su(animals) | APA PsycInfo® | 459210 |
| S16 | S1 OR S2 | APA PsycInfo® These databases are searched for part of your query. | 39453 |
| S17 | S3 OR S4 OR "S5" | APA PsycInfo® These databases are searched for part of your query. | 63473 |
| S18 | S6 OR S7 OR "S8" OR "S9" OR "S10" OR "S11" OR "S12" OR "S13" OR "S14" | APA PsycInfo® These databases are searched for part of your query. | 120428 |
| S19 | S16 AND S17 | APA PsycInfo® These databases are searched for part of your query. | 1074 |
| S20 | S18 AND S19 | APA PsycInfo® These databases are searched for part of your query. | 277 |
| S21 | S20 NOT S15 | APA PsycInfo® These databases are searched for part of your query. | 251 |

## 1.5 Cochrane

#1  MeSH descriptor: [Parkinson disease] explode all trees (4376)

#2 (aerobic exercise or aquatic exercise or balance training or body weight support treadmill or Dance Therapy or exercise* or Exercise Movement Techniques or gait training or high-speed resistance training or hydrotherapy or multicomponent exercise program or multidisciplinary exercise program or Nordic Walking or Physiotherapy or pilates or power training or Qigong or resistance training or Robotic-assisted gait training or stretch or tai ji or Tango or treadmill training or walking or Virtual Reality or whole body vibration or Yoga) in Trials (Word variations have been searched) (155706)

#3 MeSH descriptor: [resistance training] explode all trees (3641)

#4 MeSH descriptor: [exercise] explode all trees (25628)

#5 MeSH descriptor: [tai ji] explode all trees (373)

#6 MeSH descriptor: [Qigong] explode all trees (79)

#7 MeSH descriptor: [Exercise Movement Technique] explode all trees (2215)

#8 MeSH descriptor: [Yoga] explode all trees (699)

#9 MeSH descriptor: [Virtual Reality] explode all trees (284)

#10 MeSH descriptor: [hydrotherapy] explode all trees (1575)

#11 MeSH descriptor: [Dance Therapy] explode all trees (89)

#12#2 or #3 or #4 or #5 or #6 or #7 or #8 or #9 or #10 or #11 (147882)

#13#1 and #12 (906)

## 1.6 Database: Web of Science <1965 to July 20 2024>

| # 13 | 2,403 | #12 AND #11 AND #1  Indexes=SCI-EXPANDED, SSCI, A&HCI, CPCI-S, CPCI-SSH, BKCI-S, BKCI-SSH, ESCI, CCR-EXPANDED, IC Timespan=All years |  |  |
| --- | --- | --- | --- | --- |
| # 12 | 981,618 | #10 OR #9 OR #8 OR #7 OR #6 OR #5 OR #4 OR #3 OR #2  Indexes=SCI-EXPANDED, SSCI, A&HCI, CPCI-S, CPCI-SSH, BKCI-S, BKCI-SSH, ESCI, CCR-EXPANDED, IC Timespan=All years |  |  |
| # 11 | 6,290,817 | TOPIC: ((“randomized controlled trial*” or “controlled clinical trial” or “random*” or “clinical trial*” or randomly or trial or “clinical trial” or “randomized controlled trial*” or “cross-over studies” or clinic*) )  Indexes=SCI-EXPANDED, SSCI, A&HCI, CPCI-S, CPCI-SSH, BKCI-S, BKCI-SSH, ESCI, CCR-EXPANDED, IC Timespan=All years |  |  |
| # 10 | 7,671 | TOPIC: ((Yoga or “Muscle Stretching Exercises”) )  Indexes=SCI-EXPANDED, SSCI, A&HCI, CPCI-S, CPCI-SSH, BKCI-S, BKCI-SSH, ESCI, CCR-EXPANDED, IC Timespan=All years |  |  |
| # 9 | 373 | TOPIC: ((“Dance Therapy” or “Therapy, Dance” or “Dance Therapies” or “Therapies, Dance”) )  Indexes=SCI-EXPANDED, SSCI, A&HCI, CPCI-S, CPCI-SSH, BKCI-S, BKCI-SSH, ESCI, CCR-EXPANDED, IC Timespan=All years |  |  |
| # 8 | 1,197 | TOPIC: ((hydrotherapy or Hydrotherapies or “Whirlpool Baths” or “Bath, Whirlpool” or “Baths, Whirlpool” or “Whirlpool Bath”) )  Indexes=SCI-EXPANDED, SSCI, A&HCI, CPCI-S, CPCI-SSH, BKCI-S, BKCI-SSH, ESCI, CCR-EXPANDED, IC Timespan=All years |  |  |
| # 7 | 46,480 | TOPIC: (("Virtual Reality" or "Reality, Virtual" or "Virtual Reality, Educational" or "Educational Virtual Realities" or "Educational Virtual Reality" or "Reality, Educational Virtual" or "Virtual Realities, Educational" or "Virtual Reality, Instructional" or "Instructional Virtual Realities" or "Instructional Virtual Reality" or "Realities, Instructional Virtual" or "Reality, Instructional Virtual" or "Virtual Realities, Instructional") )  Indexes=SCI-EXPANDED, SSCI, A&HCI, CPCI-S, CPCI-SSH, BKCI-S, BKCI-SSH, ESCI, CCR-EXPANDED, IC Timespan=All years |  |  |
| # 6 | 234 | TOPIC: (("Exercise Movement Techniques" or "Movement Techniques, Exercise" or "Exercise Movement Technics" or "Pilates-Based Exercises" or "Exercises, Pilates-Based" or "Pilates Based Exercises" or "Pilates Training" or "Training, Pilates"）)  Indexes=SCI-EXPANDED, SSCI, A&HCI, CPCI-S, CPCI-SSH, BKCI-S, BKCI-SSH, ESCI, CCR-EXPANDED, IC Timespan=All years |  |  |
| # 5 | 4,058 | TOPIC: (“Tai-ji” or “Tai Chi” or “Chi, Tai” or “Tai Ji Quan” or “Ji Quan, Tai” or “Quan, Tai Ji” or Taiji or Taijiquan or “T'ai Chi” or “Tai Chi Chuan” Qigong or “Qi Gong” or “Ch'i Kung”)  Indexes=SCI-EXPANDED, SSCI, A&HCI, CPCI-S, CPCI-SSH, BKCI-S, BKCI-SSH, ESCI, CCR-EXPANDED, IC Timespan=All years |  |  |
| # 4 | 625,989 | TOPIC: (Exercise* or “Exercise Program, Weight-Bearing” or “Exercise Programs, Weight-Bearing” or “Weight Bearing Exercise Program” or “Weight-Bearing Exercise Programs” Exercise* or “Physical Activity” or “Activities, Physical” or “Activity, Physical” or “Physical Activities” or “Exercise, Physical” or “Exercises, Physical” or “Physical Exercise” or “Physical Exercises” or “Exercise, Isometric” or “Exercises, Isometric” or “Isometric Exercises” or “Isometric Exercise” or “Exercise, Aerobic” or “Aerobic Exercise” or “Aerobic Exercises” or “Exercises, Aerobic” or “Exercise Training” or “Exercise Trainings” or “Training, Exercise” or “Trainings, Exercise”)  Indexes=SCI-EXPANDED, SSCI, A&HCI, CPCI-S, CPCI-SSH, BKCI-S, BKCI-SSH, ESCI, CCR-EXPANDED, IC Timespan=All years |  |  |
| # 3 | 17,738 | TOPIC: ("Resistance training” or “Training, Resistance” or “Strength Training” or “Training, Strength” or “Weight-Lifting Strengthening Program” or “Strengthening Program, Weight-Lifting” or “Strengthening Programs, Weight-Lifting” or “Weight Lifting Strengthening Program” or “Weight-Lifting Strengthening Programs” or “Weight-Lifting Exercise Program” or “Exercise Program, Weight-Lifting” or “Exercise Programs, Weight-Lifting” or “Weight Lifting Exercise Program” or “Weight-Lifting Exercise Programs” or “Weight-Bearing Strengthening Program” or “Strengthening Program, Weight-Bearing” or “Strengthening Programs, Weight-Bearing” or “Weight Bearing Strengthening Program” or “Weight-Bearing Strengthening Programs” or “Weight-Bearing Exercise Program”)  Indexes=SCI-EXPANDED, SSCI, A&HCI, CPCI-S, CPCI-SSH, BKCI-S, BKCI-SSH, ESCI, CCR-EXPANDED, IC Timespan=All years |  |  |
| # 2 | 424,147 | TOPIC: (“aerobic exercise” or “aquatic exercise” or “balance training” or “body weight support treadmill” or “Dance Therapy or exercise*” or “Exercise Movement Techniques” or “gait training” or “high-speed resistance training” or “hydrotherapy” or “multicomponent exercise program” or “multidisciplinary exercise program” or “Nordic Walking” or “Physiotherapy” or pilates or “power training” or Qigong or “resistance training” or “Robotic-assisted gait training” or stretch or “tai ji” or Tango or “treadmill training” or “walking” or “Virtual Reality” or “whole body vibration” or Yoga)  Indexes=SCI-EXPANDED, SSCI, A&HCI, CPCI-S, CPCI-SSH, BKCI-S, BKCI-SSH, ESCI, CCR-EXPANDED, IC Timespan=All years |  |  |
| # 1 | 113,262 | TOPIC: ("Idiopathic Parkinson's Disease" or "Lewy Body Parkinson's Disease" or "Parkinson's Disease, Idiopathic" or "Parkinson's Disease, Lewy Body" or "Parkinson Disease, Idiopathic" or "Parkinson's Disease" or "Idiopathic Parkinson Disease" or "Lewy Body Parkinson Disease" or "Primary Parkinsonism" or "Parkinsonism, Primary" or "Paralysis Agitans")  Indexes=SCI-EXPANDED, SSCI, A&HCI, CPCI-S, CPCI-SSH, BKCI-S, BKCI-SSH, ESCI, CCR-EXPANDED, IC Timespan=All years |  |  |

# **Supplementary File 2: Definitions of exercise types and non-exercise training control**

| **Abbreviation** | **Full name** | **Definitions** |
| --- | --- | --- |
| AE | Aerobic Exercise | Aerobic exercise is performed by repeating sequences of light-to-moderate intensity activities for extended periods.^1^ e.g., walking, bicycle, and treadmill training, etc. |
| AQE | Aquatic Exercise | Gait training, balance training, resistance training, or aerobic training performed in deep or shallow water.^2^ |
| BGT | Balance and Gait Training | Single-task balance and gait training without external cues or internal and external attention |
| CON | Control group | Non-exercise intervention, usual care,^3^ or health education |
| Dance |  | Group dances other than tango, such as waltz, Irish set dancing, etc. |
| MulC | Mixed Exercise Program | Two or more of the above specific types of exercise training (if it is only part of warm-up or relaxation, it is not considered as multi-mode) |
| RT | Resistance Training | Exercise training is designed to improve the strength, power, endurance, and size of skeletal muscles.^4^ |
| SE | Sensory Exercise | Focus on ‘internal’ or ‘external sensory’ feedback while doing balance and gait training.^5^ |
| **MBE (Mind Body Exercise)** | | |
| TC | Tai Chi | It is an internal Chinese martial art practiced for defense training, health benefits, and meditation. |
| Yoga |  | Mainly a series of methods for self-cultivation, including body-adjusting asanas (refer to yoga asana collection), breathing-adjusting breathing methods, and mind-adjusting meditation, etc., to achieve the unity of body and mind.^6^ |
| Qigong |  | It is a system of coordinated body posture and movement, breathing, and meditation used for health, spirituality, and martial arts training. |

**Reference**

1. Plowman SA, Smith DL. Exercise physiology for health fitness and performance: Lippincott Williams & Wilkins; 2013.

2. Konlian C. Aquatic therapy: making a wave in the treatment of low back injuries. Orthop Nurs 1999; 18(1).

3. Goh S-L, Persson MSM, Stocks J, et al. Relative Efficacy of Different Exercises for Pain, Function, Performance and Quality of Life in Knee and Hip Osteoarthritis: Systematic Review and Network Meta-Analysis. Sports Med 2019; 49(5): 743-61.

4. Powell KE, Paluch AE, Blair SN. Physical activity for health: What kind? How much? How intense? On top of what? Annu Rev Public Health 2011; 32: 349-65.

5. Abdollahipour R, Wulf G, Psotta R, Palomo Nieto M. Performance of gymnastics skill benefits from an external focus of attention. J Sports Sci 2015; 33(17): 1807-13.

6. Cramer H, Lauche R, Haller H, Dobos G. A systematic review and meta-analysis of yoga for low back pain. Clin J Pain 2013; 29(5): 450-60.

# **Supplementary File 3: Table 1: Characteristics of included studies**

| **Author** | **Age**  **(Mean±SD)** | **Number**  **(men)** | **Years of diagnosis( Mean±SD)** | **Hoehn and Yahr stage (Mean±SD)** | **Follow Up** | **Duration (weeks)** | **Frequency** | **Time**  **(minutes)** | **Overall balance** |
| --- | --- | --- | --- | --- | --- | --- | --- | --- | --- |
| Martin Benka Wallén1 et al.[1] | SE: 73.1±5.8  CON: 73.0±5.5 | SE: 51(32) CON: 49(25) | SE: 5.9±5.1 CON: 5.6±4.8 | SE: 2.55±0.5 CON: 2.57±0.5 | OFF | 10 | 3 | 60 | Mini-BESTest, score |
| Petra Pohl et al[2] | SE: 69.7 ± 7.0 CON: 70.4 ± 6.0 | SE: 26(19) CON: 20(13) | SE: 6.0 ± 4.4 CON: 6.8 ± 3.6 | SE: 2.4±0.69 CON: 2.3±0.65 | ON | 12 | 2 | 60 | Mini-BESTest, score |
| Peter S. Myers et al.[3] | CON: 65.0±8.7 MBE: 70.5±8.7 | CON: 13(8) MBE: 13(7) | NA | CON:2(2-3) MBE:2(2-3) | OFF | 12 | 2 | 60 | BESTest, score |
| suhaila M. saNtos et al.[4] | BGT: 68.5±6.5  RT: 67.0±7.9 | BGT: 21(7) RT: 19(11) | BGT: 5.4±5.3 RT: 5.6±4.2 | BGT: 2.3±0.6 RT: 2.3±0.5 | ON | 8 | 2 | 60 | BESTest, score |
| Marianna Capecci et al.[5] | BGT: 66.8±4.9 CON: 68.1±5.6 | BGT: 7(4) CON: 7(4) | BGT: 9.5±7.4 CON: 9.6±4.9 | BGT: 3.3±0.7 CON: 3.3±0.9 | OFF | 4 | 3 | 40 | BBS, score |
| Nicholas P. Cherup et al.[6] | MBE: 69.8±7.3   BGT: 71.4±12.1 | MBE: 15(10)  BGT: 18(11) | NA | MBE: 1.7±0.5 BGT: 2±0.8 | ON | 12 | 2 | 45 | Tinetti assessment scale, score |
| David CoNAadsson et al.[7] | SE: 72.9±6.0 CON: 73.6±5.3 | SE: 47 (28)  CON: 44(23) | SE: 6.0±5.1 CON: 5.6±5.0 | SE: 2.6±0.5 CON: 2.6±0.5 | OFF | 10 | 3 | 60 | Mini-BESTest |
| Marialuisa Gandolfi et al.[8] | SE: 67.5±7.2 BGT: 69.8±9.4 | SE: 38(23) BGT: 38(28) | SE: 6.2±3.8 BGT: 7.5±3.9 | 2.5 | OFF | 7 | 3 | 50 | BBS, score |
| Madeleine E. Hackney et al.[9] | MBE: 64.9± 8.3 CON: 62.6±10.2 | MBE: 17(11) CON: 15(10) | MBE: 8.7±4.7 CON: 5.5±3.3 | MBE: 2±0.4 CON: 1.9± 0.2 | ON | 13 | 2 | 60 | BBS, score |
| Arva Khuzema et al.[10] | MBE: 72±5.22 BGT: 70.89±6.01 | MBE: 9(6) BGT: 9(7) | MBE: 5.67±2.33 BGT: 5.23±3.12 | MBE: 2.83±0.24 BGT: 2.78±0.25 | ON | 8 | 5 | 35 | BBS, score |
| Meng Ni et al[11] | RT: 71.6±6.6 MBE: 71.2±6.5 CON: 74.9± 8.3 | RT: 14(9) MBE: 13(11) CON: 10(4) | RT: 6.6±4.4 MBE: 6.9±6.3 CON: 5.9±6.2 | RT: 2.2±0.6 MBE: 2.2±0.7 CON: 2.1± 0.7 | ON | 12 | 2 | 60 | BBS, score |
| Christian Schlenstedt et al[12] | RT: 75.7 ± 5.5 BGT: 75.7 ± 7.2 | RT:17(12) BGT: 15(9) | RT: 10.1 ± 6.0 BGT: 9.3 ± 7.9 | RT: 2.8 ± 0.26 BGT: 2.7 ± 0.4 | ON | 7 | 2 | 60 | Fullerton Advanced Balance, score |
| Camila Gemin Ribas et ail[13] | SE: 61.7±6.8 BGT: 60.2±11.2 | SE: 10 (4) BGT: 10 (4) | SE: 6.5±4 BGT: 7±2.8 | SE: 1.4±0.5 BGT: 1.5±0.5 | ON | 12 | 2 | 30 | BBS, score |
| Pietro Santosa et al.[14] | SE: 61.7±7.3 RT: 64.5±9.8 SE: 66.6±8.2 | SE: 13(11) RT: 14(11) SE: 14(9) | SE: 7±2.8 RT: 6.5±2.0 SE: 7.8±3.7 | SE: 1.4±0.6 RT: 1.3±0.3 SE: 1.5±0.4 | ON | 8 | 2 | 50 | BBS, score |
| Simon Steib, PhD et al.[15] | SE: 67.5 ±8.2 AT: 62.5±7.9 | SE: 18(11) AT: 20(16) | SE: 7.9±4.0 AT: 7.3±4.4 | SE: 2.6±0.5 AT: 2.5±0.5 | ON | 8 | 2 | 40 | Mini-BESTest |
| Chun-Mei Xiao[16] | CON: 66.5±2.1 MBE: 68.1±2.3 | CON: 48(34) MBE: 48(33) | CON: 6.2±2.6 MBE: 5.5±3.6 | CON: 2.1±0.2 MBE: 2.2±0.2 | ON | 24 | 4 | 50 | BBS, score |
| Chunmei Xiao et al.[17] | 67.8±9.4 | MBE: 49 CON: 49 | NA | NA | ON | 24 | 4 | 60 | BBS, score |
| Wen-Chieh Yang et al.[18] | SE: 72.5±8.4 BGT: 75.4±6.3 | SE: 11(7) BGT: 12(7） | SE: 9.4±3.6 BGT: 8.3±4.1 | SE: 3±0 BGT: 3±0 | OFF | 6 | 2 | 50 | BBS, score |
| Tian-Yu Zhang, BS et al.[19] | MBE: 66.0±11.8 Mul_C: 64.4±10.5 | MBE: 20(13) Mul_C: 20(11) | MBE: 6.8±5.4 Mul_C: 4.9±3.7 | MBE: 2.0±0.5 Mul_C: 2.2±0.4 | ON | 12 | 2 | 60 | BBS, score |
| Hasan Daneshmandi[20] | MBE: 57±6.2 CON: 58.3±7.4 | MBE: 15(8) CON: 15(10) | MBE: 7.3±3.8 CON: 8.2±3.1 | MBE: 2.7±0.5 CON: 2.6±0.5 | ON | 8 | 3 | 60 | Fullerton Advanced Balance, score |
| Meng-Che Shih[21] | SE: 67.5±10.0 BGT: 68.8±9.7 | SE: 10(9) BGT: 10(7) | SE: 4.0±3.7 BGT: 5.2±4.9 | SE: 1.6±0.8 BGT: 1.4±0.5 | ON | 8 | 2 | 50 | BBS, score |
| Rocco Salvatore Calabrò et al[22] | SE: 70±8 AE: 73±8 | SE: 25(11) AE: 25(14) | SE: 10.0±3.0 AE: 9.3±3.0 | SE: 3.0±1.0 AE: 3.0±1.0 | OFF | 8 | 5 | 25 | BBS, score |
| Amit Abraham[23] | SE:66.4±12.5 CON: 65.1±7.5 | SE:10(9) CON:10(7) | SE: 6.1±3.8 CON: 8.5±4.5 | SE: 2.0±0.52 CON: 2.0±0.37 | OFF | 2 | 5 | 120 | Mini-BESTest |
| Alessandro Carvalho[24] | AE: 64.8±11.9 RT: 64.1±9.9 BGT: 62.1±11.7 | AE: 5(4) RT: 8(6) BGT: 9(5) | AE: 6.6±1.5 RT: 6.0±2.6 BGT: 4.3±2.8 | AE: 2.6±0.5 RT: 2.1±0.6 BGT: 2.3±0.5 | OFF | 12 | 2 | 40 | BBS, score |
| Ilaria Arcolin[25] | AE: 67.8±8.8 AE: 68.7±8.3 | AE: 13(6) AE: 16(9) | AE: 6.5±2.9 AE: 4.7±2.9 | AE: 2.3±0.5 AE: 2.3±0.5 | OFF | 3 | 5 | 60 | mini-BESTest, score |
| Tuğba ATAN et al.[26] | AE: 69.7±8 AE: 72.2±7.9 AE: 68.6±8.2 | AE: 10(3) AE: 10(4) AE: 10(4) | AE: 69.7±8 AE: 72.2±7.9 AE: 68.6±8.2 | AE: 2.6±0.7 AE: 2.8±0.6 AE: 2.7±0.7 | ON | 6 | 5 | 30 | BBS, score |
| Dae-Hyouk Bang[27] | AE: 58.30±7.71 AE: 60.60±6.74 | AE: 10(5) AE: 10(4) | months AE: 18.10±6.77 AE: 17.98±3.28 | AE: 2.32±0.52 AE: 2.56±0.51 | ON | 4 | 5 | 60 | BBS, score |
| Ilaria Carpinella et al[28] | SE: 73.0±7.1 CON: 75.6±8.2 | SE: 17(14) CON: 20(9) | SE: 7.5±3.2 CON: 10.3±5.7 | SE: 12.7±0.7 CON: 2.9±0.5 | ON | 7 | 3 | 45 | BBS, score |
| Kim Chivers Seymour et al[29] | Mul_C: 71 ±7.7 CON: 73 ±7.7 | Mul_C: 238(147) CON: 236(119) | Mul_C: 8±6.6 CON: 8±5.8 | Mul_C: 2.59±2.67 CON: 0.86±0.89 | ON | 26 | 7 | 30 | Mini-BESTest, score |
| Silvia Rios Romenets et al.[30] | CON: 64.3±8.1 Dance: 63.2±9.9 | CON: 15(7) Dance: 18(12) | CON 7.7±4.6 Dance: 5.5±4.4 | CON: 2.0±0.5 Dance: 1.7±0.6 | ON | 12 | 2 | 60 | Mini-BESTest, score |
| Lucia Cugusi et al.[31] | AE: 68.1 ± 8.7 CON: 66.6±7.3 | AE: 10 (8) CON: 10(8) | AE: 7±2 CON: 7±4 | AE: 2.4 ± 0.8 CON: 2.3 ± 0.5 | ON | 12 | 2 | 60 | BBS, score |
| G.Frazzitta et al.[32] | SE: 66.6±10.0  AE: 65.0±8.8 | SE: 30(13) AE: 30(17) | NA | SE: 2.8 ±0.4 AE: 2.8±0.4 | ON | 4 | 6 | 35 | BBS, score |
| Pieter Ginis et al.[33] | NA | SE: 20 BGT: 18 | NA | 2-3 | ON | 6 | 3 | 30 | Mini-BESTest, score |
| Victoria A Goodwin et al.[34] | Mul_C: 72.0±8.6 CON: 70.1±8.3 | Mul_C: 64(39) CON: 66(35) | Mul_C: 9.1±6.4 CON 8.2±6.4 | Mul_C: 2.6 ±0.9 CON: 2.4 ±0.9 | ON | 10 | 1 | 60 | BBS, score |
| Madeleine E. Hackney et al. [35] | Dance: 72.6±2.2 RT: 69.6±2.1 | Dance: 9(6) RT: 10(6) | Dance: 6.2±1.5 RT: 3.3±0.5 | Dance: 2.3±0.7 RT: 2.2±0.6 | ON | 13 | 2 | 60 | BBS, score |
| C.C. Harro et al. [36] | SE: 67.3±10.9 AE: 64.9±9.0 | SE: 10(8) AE: 10(5) | SE: 4.0±2.1 AE: 4.3±2.3 | SE: 1.9±0.5 AE: 2.0±0.6 | ON | 6 | 3 | 30 | BBS, score |
| Adriano Zanardi da Silva [37] | AQE: 63.12 ± 13.61 CON: 64.23 ± 13.45 | AQE: 14(6) CON: 11(5) | NA | AQE: 3±1 CON: 3±1 | ON | 10 | 2 | 60 | BBS, score |
| József Tollára [38] | SE: 70.0±4.69 AE: 70.6±4.10 CON: 67.5±4.28 | SE: 25(12) AE: 25(11) CON: 24(13) | SE: 7.5±1.76 AE: 7.5±2.16 CON: 7.3±2.21 | SE: 2.3±0.48 AE: 2.4±0.51 CON: 2.4±0.51 | OFF | 5 | 5 | 60 | Mini-BESTest, score |
| José Eduardo Pompeu et al.[39] | 67.4±8.1 | 32(17) | NA | 1.7±0.5 | ON | 7 | 2 | 60 | BBS, score |
| Irene S.K. Wong-Yu et al.[40] | Mul_C: 59.4±9.0 CON: 62.6±8.9 | Mul_C: 41(25) CON: 39(21) | Mul_C: 7.1±4.3 CON: 5.6±3.8 | Mul_C: 2.5±0.3 CON: 2.4±0.3 | ON | 8 | 1 | 120 | BESTest total, score |
| Daniele Volpe et al.[41] | SE: 66.5±10.4 BGT: 69.5±6.5 | SE: 20(7) BGT: 20(9) | SE: 6.0±5.0 BGT: 6.5±3.7 | SE: 3.0±0.0 BGT: 3.0±0.7 | ON | 8 | 5 | 60 | BBS score |
| Zahra Kadivar[42] | SE: 73.3±2.2 BGT: 70.5±2.2 | SE: 8(5) BGT: 8(6) | SE: 8.9±1.8 BGT: 7.5±1.2 | SE: 2.69±0.56 BGT: 2.69±0.56 | OFF | 6 | 3 | 50 | Tinetti-gait and balance tests, Tinetti balance |
| Bakhshayesh Babak[43] | MBE: 57.0±6.24  BGT: 58.31±7.37 | 30(18) | MBE: 7.27±3.80  BGT: 8.19±3.14 | 2-3 | ON | 8 | 3 | 60 | Fullerton advanced balance score |
| Paolo Solla[44] | Dance: 67.8±5.9 CON: 67.1±6.3 | Dance: 10(6) CON: 10(7) | Dance: 4.4±4.5 CON: 5.0±2.9 | Dance: 2.1±0.6 CON: 2.3±0.4 | ON | 12 | 2 | 90 | BBS, score |
| Kristi Michels[45] | Dance: 66.44±NA CON: 75.50±NA | Dance: 9(NA) CON: 4(NA) | NA | Dance: 2.11±0.33 CON: 2.50±1.00 | ON | 10 | 2 | 60 | BBS, score |
| D Kunkel[46] | Dance: 71.3±7.7 CON: 69.7±6.0 | Dance: 36(19) CON: 15(6) | Dance: 4.7±3.5 CON: 7.0±4.9 | Dance: 2.11±0.84 CON: 2.13±0.72 | ON | 10 | 2 | 60 | BBS, score |
| Nicola Smania[47] | SE: 67.64±7.41 CON: 67.26±7.18 | SE: 28（14） CON:27（15） | SE: 10.39±4.76 CON: 8.63±5.39 | SE: 14.6±5.9 CON: 3.1±0.3 | ON | 7 | 3 | 50 | BBS, score |
| Hanan Khalil et al[48] | Mul_C: 58.4±13.5 CON: 60.7±15.4 | Mul_C: 16(12) CON: 14(7) | Mul_C: 8.0±6.4 CON: 7.5±4.0 | Mul_C: 2.4±0.72 CON: 2.2±0.8 | ON | 8 | 3 | 45 | Mini-BESTest, score |
| Merrill R Landers[49] | SE: 72.2±4.4 SE: 70.2±4.4 BGT: 70.1± 9.5 CON: 74.3±8.8 | SE: 10(4) SE: 11(8) BGT: 10(7) CON: 10(6) | NA | SE: 2.25±0.86 SE: 2.75±0.75 BGT: 2.45±0.44 CON: 2.75±0.63 | OFF | 4 | 3 | 45 | BBS, score |
| Emine Eda Kurt et al[50] | AQE: 62.41 ± 6.76 Mul_C: 63.61 ± 7.18 | AQE: 20(11) Mul_C: 20(13) | NA | AQE: 2.37±0.39 Mul_C: 2.32±0.40 | OFF | 5 | 5 | 60 | BBS, score |
| Leon CP Leal et al[51] | CON: 64.9±2.32  RT: 65.2±2.05 | CON: 27(13)  RT: 27(14) | NA | CON: 2±0.5  RT: 2±0.5 | OFF | 24 | 2 | 32.5 | Tinetti mobility test, score |
| Hwa-Jin Lee, PhD et al.[52] | MBE: 65.8±7.2 CON: 65.7±6.4 | MBE: 25(10) CON: 16(7) | MBE: 4.5±3.3 CON: 4.4±3.0 | MBE: 2.0±0.7 CON: 1.8±0.8 | OFF | 8 | 2 | 60 | BBS, score |
| Araceli Ortiz-Rubio et al[53] | RT: 74.2±5.8 CON: 75.4±6.5 | RT: 23(NA) CON: 23(NA) | RT: 4.0±2.2 CON: 4.3±2.0 | RT: 2.5±0.5 CON: 2.4±0.5 | ON | 8 | 2 | 60 | Mini-BESTest, score |
| Grazia Palamara et al.[54] | AQE: 70.9±5.7 Mul_C: 70.8±5.3 | AQE: 17(9) Mul_C: 17(11) | NA | AQE: 2.8±0.5 Mul_C: 3.1±0.2 | ON | 4 | 4 | 60 | BBS, score |
| A. Park et al[55] | RT: 60.1±6.6 CON: 59.8±6.3 | RT: 15(10) CON: 16(10) | NA | NA | ON | 48 | 3 | 60 | Tinetti Mobility Test |
| Carla Silva-Batista et al. [56] | CON: 64.2±8.3 Mul_C: 64.1±9.1  RT: 64.2±10.6 | CON: 13(9) Mul_C: 13(10)  RT: 13(10) | CON: 10.7±6.1 Mul_C: 9.6±3.9  RT: 10.5±4.1 | CON: 2.5±0.4 Mul_C: 2.5±0.5  RT: 2.5±0.4 | OFF | 12 | 2 | 60 | BESTest total score（%） |
| Maarten R.C. van den Heuvel et al.[57] | SE: 66.3±6.39 SE: 68.8±9.68 | SE: 17(12) SE: 16(8) | SE: 9.0±2.3 SE: 8.8±2.3 | SE: 2.5±0.4 SE: 2.5±0.4 | ON | 5 | 2 | 60 | BBS, score |
| Daniele Volpe1 et al.[58] | AQE: 68 ± 7 BGT: 66 ± 8 | AQE:17(NA) BGT:17(NA) | AQE: 7.5 ± 5.1 BGT: 7.6 ± 4.63 | AQE: 2.82 ± 0.3 BGT: 2.65 ± 0.49 | ON | 8 | 5 | 60 | BBS, score |
| Daniele Volpe1 et al.[59] | AQE: 70.6 ± 7.8  Mul_C: 70 ± 7.8 | AQE: 15(9) Mul_C: 15(10) | AQE: 9.4 ± 7.5  Mul_C: 9 ± 7.0 | AQE: 2.6 ± 0.5 Mul_C: 2.7 ± 0.5 | ON | 8 | 5 | 60 | BBS, score |
| Irene Cabrera-Martos et al.[60] | RT: 77.2±6.2 CON: 75.9±1.2 | RT: 22(15) CON: 22(11) | NA | 2-3 | OFF | 8 | 3 | 45 | Mini-BESTest, score |
| José Ma Cancela [61] | AQE: 67.7±4.6 Mul_C: 69.2±4.4 | AQE: 7(6) Mul_C: 5(3) | NA | AQE: 2.3±0.8 Mul_C: 2.2±0.5 | ON | 8 | 3 | 50 | Tinetti assessment scale, score |
| Tamine T.C. Capato et al.[62] | SE: 74±8 BGT: 67±13 CON: 73±10 | SE: 56(27) BGT: 50(32) CON: 48(29) | SE: 5±5.2 BGT: 6±5.9 CON: 8±9.6 | SE: 2.3±0.8 BGT: 2.2±0.8 CON: 2.3±0.7 | ON | 5 | 2 | 45 | Mini-BESTest, score |
| Tamine T.C. Capato et al.[63] | SE: 77±7 BGT: 78±10 | SE: 17(9) BGT: 18(12) | SE: 17±9 BGT: 11±4 | NA | ON | 5 | 2 | 45 | BBS, score |
| Qiang Gao et al.[64] | MBE: 69.5±7.3 CON: 68.3±8.5 | MBE: 37(23) CON: 39(27) | MBE: 9.2±8.6 CON: 8.4±8.2 | MBE: 2.4±0.5 CON: 2.4±0.7 | OFF | 12 | 3 | 60 | BBS, score |
| Hanna Johansson et al.[65] | SE: 72±13.3 CON: 67.5±5.2 | SE: 7(6)  CON: 6(3) | SE: 10±7.4 CON: 7±5.9 | SE: 2±NA CON: 2.5±NA | OFF | 10 | 2 | 60 | Mini-BESTest, score |
| Conran Joseph et al.[66] | SE: 73.1±5.8  CON: 73.0±5.5 | SE: 51(32) CON: 49(25) | SE: 5.9±5.1 CON: 5.6±4.8 | SE: 2.55±0.5 CON: 2.57±0.5 | OFF | 10 | 3 | 60 | Mini-BESTest, score |
| L. A. King al.[67] | Mul_C:65.7 ± 8.3 AE: 65.1 ± 7.3 | Mul_C: 20(12) AE: 19(13) | NA | Mul_C: 2.5 ± 0.8 AE: 2.4 ± 0.6 | ON | 4 | 4 | 75 | mini-BESTest, score |
| Se Hee Jung al.[68] | Mul_C: 67.7±6.7 CON: 70.0±8.2 | Mul_C: 44(30) CON: 42(28) | Mul_C: 6.2±4.4 CON: 6.7±5.5 | Mul_C: 2.11±0.44 CON: 2.42±0.76 | ON | 6 | 3 | 80 | mini-BESTest, score |
| Christian Schlenstedt et al[69] | RT: 78.3±5.8 BGT: 81.4±7.3 | RT: 12(9) BGT: 8(6) | RT: 11.2±6.6 BGT: 8.4 ±7.3 | RT: 2.8±0.3 BGT: 2.9±0.5 | ON | 7 | 2 | 60 | Fullerton Advanced Balance, score |
| Esther M. J. Bekkers, PhD et al.[70] | AE: 70.9±6.0 VR: 71.1±6.3 | AE: 59(37) VR: 62(37) | AE: 9.6±7.2 VR: 9.1±5.5 | AE: 2.5±0.5 VR: 2.4±0.5 | OFF | 6 | 3 | 45 | mini-BESTest, score |
| Hsin-Hsuan Liu, PT et al.[71] | BGT: 70.93±7.23 CON: 64.79±5.86 | BGT: 14(8) CON: 14(8) | BGT: 6.82 ± 3.94 CON: 6.96 ± 6.27 | BGT: 1.96 ± 0.72 CON: 1.54 ± 0.72 | ON | 8 | 2 | 60 | Mini BBS, score |
| Zhenlan Li et al[72]. | MBE：67.57±3.95 CON: 70±5.59 | MBE：20(13) CON: 20(16) | MBE：6.83 ± 4.09 CON:7.76 ± 4.55 | MBE：1.5±1.5 CON: 1.6±0.59 | ON | 12 | 2 | 60 | Mini BBS, score |
| Washington University School of Medicine | AE: 68.52±9.54 Dance: 66.73±9.52 CON: 66.18±7.3 | AE: 31(17) Dance: 39(25) CON: 26(14) | AE: 5.59±3.81 Dance: 6.10±4.82 CON: 4.4±5.04 | NA | ON | 12 | 2 | 60 | Mini-BESTest |

**N/A not available,** **AE Aerobic Exercise, AQE Aquatic Exercise, BGT: Balance and Gait Training, CON: Control group, Mul_C: Multi-component Exercise Program, RT Resistance Training, Dance; MBE: Mind-body Exercise, SE Sensory Exercise, BBS: Berg Balance Scale, Mini-BESTest: Balance Evaluation Systems Test;**

## **Table 2: Inclusion of study-specific data**

| **Author** | **Agent** | **Dose** | **Mean^change^** | **SE** | **N** | **MET** | **Redidual dose** | **MET-min/week** | **Total MET** | **Time** | **Frequency** | **Period** |
| --- | --- | --- | --- | --- | --- | --- | --- | --- | --- | --- | --- | --- |
| Martin Benka Wallén1 et al. | CON | 0 | 1.1 | 0.516661181 | 49 | 0 | 0 | 0 | 0 | 0 | 0 | 0 |
|  | SE | 500 | 2.9 | 0.420783583 | 51 | 2.5 | -50 | 450 | 4500 | 60 | 3 | 10 |
| Petra Pohl et al | CON | 0 | -0.3 | 1.259595173 | 15 | 0 | 0 | 0 | 0 | 0 | 0 | 0 |
|  | SE | 250 | -0.6 | 0.994868836 | 25 | 2.3 | 26 | 276 | 3312 | 60 | 2 | 12 |
| Peter S. Myers et al. | CON | 0 | 2 | 2.080125736 | 13 | 0 | 0 | 0 | 0 | 0 | 0 | 0 |
|  | MBE | 250 | 3 | 2.246535795 | 13 | 2.5 | 50 | 300 | 3600 | 60 | 2 | 12 |
| suhaila M. saNtos et al. | RT | 500 | -0.9 | 1.705100707 | 19 | 3.5 | -80 | 420 | 3360 | 60 | 2 | 8 |
|  | BGT | 250 | 3 | 1.289702808 | 21 | 2.3 | 26 | 276 | 2208 | 60 | 2 | 8 |
| Marianna Capecci et al. | CON | 0 | -1 | 0.629058253 | 7 | 0 | 0 | 0 | 0 | 0 | 0 | 0 |
|  | BGT | 250 | 6 | 2.060859737 | 7 | 2.3 | 26 | 276 | 1104 | 40 | 3 | 4 |
| Nicholas P . Cherup et al. | BGT | 250 | 0.5 | 0.954812372 | 18 | 2.3 | -43 | 207 | 2484 | 45 | 2 | 12 |
|  | MBE | 250 | 2.2 | 0.536656315 | 15 | 2.5 | -25 | 225 | 2700 | 45 | 2 | 12 |
| David CoNAadsson et al. | CON | 0 | 0.9 | 0.500508832 | 44 | 0 | 0 | 0 | 0 | 0 | 0 | 0 |
|  | SE | 500 | 3 | 0.500316921 | 47 | 3 | 40 | 540 | 2160 | 60 | 3 | 4 |
| Marialuisa Gandolfi et al. | BGT | 250 | 4.21 | 1.153734212 | 38 | 2.3 | 95 | 345 | 2415 | 50 | 3 | 7 |
|  | SE | 500 | 3.74 | 0.886748941 | 38 | 3.8 | 70 | 570 | 3990 | 50 | 3 | 7 |
| Madeleine E. Hackney et al. | CON | 0 | -0.5 | 2.099540243 | 13 | 0 | 0 | 0 | 0 | 0 | 0 | 0 |
|  | MBE | 250 | 3.3 | 3.000928062 | 13 | 3 | 110 | 360 | 4680 | 60 | 2 | 13 |
| Arva Khuzema et al. | MBE | 500 | 8.4 | 2.12773223 | 9 | 3 | 25 | 525 | 4200 | 35 | 5 | 8 |
|  | BGT | 500 | 6.333 | 2.847671563 | 9 | 2.3 | -97.5 | 402.5 | 3220 | 35 | 5 | 8 |
| Meng Ni et al | CON | 0 | 0.4 | 1.928989373 | 10 | 0 | 0 | 0 | 0 | 0 | 0 | 0 |
|  | MBE | 250 | 4.2 | 1.913715677 | 13 | 2.5 | 50 | 300 | 3600 | 60 | 2 | 12 |
|  | RT | 500 | 4.4 | 1.518043854 | 14 | 5 | 100 | 600 | 7200 | 60 | 2 | 12 |
| Christian Schlenstedt et al | RT | 500 | 2.3 | 1.243335173 | 17 | 3.5 | -80 | 420 | 2940 | 60 | 2 | 7 |
|  | BGT | 250 | 0.4 | 1.287633488 | 15 | 2.3 | 26 | 276 | 1932 | 60 | 2 | 7 |
| Camila Gemin Ribas et ail | BGT | 250 | -0.2 | 0.875688301 | 10 | 2.3 | -112 | 138 | 1656 | 30 | 2 | 12 |
|  | SE | 250 | 1.9 | 0.811560226 | 10 | 3.8 | -22 | 228 | 2736 | 30 | 2 | 12 |
| Pietro Santosa et al. | RT | 0 | 5.1 | 1.470665544 | 14 | 2.3 | -112 | 138 | 1656 | 30 | 2 | 12 |
|  | SE | 250 | 5.3 | 1.362407372 | 13 | 3.8 | -22 | 228 | 2736 | 30 | 2 | 12 |
|  | RT | 0 | 5.1 | 1.470665544 | 14 | 2.3 | -112 | 138 | 1656 | 30 | 2 | 12 |
|  | SE | 250 | 5.5 | 1.443210706 | 14 | 3.8 | -22 | 228 | 2736 | 30 | 2 | 12 |
| Simon Steib, PhD et al. | SE | 250 | 0.6 | 0.565685425 | 18 | 2.5 | -50 | 200 | 1600 | 40 | 2 | 8 |
|  | AE | 250 | -0.3 | 0.335410197 | 20 | 2.3 | -66 | 184 | 1472 | 40 | 2 | 8 |
| Chun-Mei Xiao | CON | 0 | -1.2 | 2.087972048 | 44 | 0 | 0 | 0 | 0 | 0 | 0 | 0 |
|  | MBE | 500 | 2.5 | 1.445990625 | 45 | 3 | 100 | 600 | 14400 | 50 | 4 | 24 |
| Chunmei Xiao, Med et al. | CON | 0 | 4.4 | 0.665092839 | 33 | 0 | 0 | 0 | 0 | 60 | 4 | 24 |
|  | MBE | 750 | 5.1 | 1.150310517 | 35 | 3 | -30 | 720 | 17280 | 60 | 4 | 24 |
| Wen-Chieh Yang et al. | BGT | 250 | 4.2 | 1.812686772 | 12 | 2.3 | -20 | 230 | 1380 | 50 | 2 | 6 |
|  | SE | 500 | 3.4 | 1.816840414 | 11 | 3.8 | -120 | 380 | 2280 | 50 | 2 | 6 |
| Tian-Yu Zhang, BS et al. | Mul_C | 500 | 2 | 0.487462819 | 20 | 4.75 | 70 | 570 | 6840 | 60 | 2 | 12 |
|  | MBE | 250 | 1.45 | 0.724486025 | 20 | 3 | 110 | 360 | 4320 | 60 | 2 | 12 |
| Hasan Daneshmandi | CON | 0 | 0.26 | 1.316814338 | 15 | 0 | 0 | 0 | 0 | 0 | 0 | 0 |
|  | MBE | 500 | 5.67 | 1.722186595 | 15 | 3 | 40 | 540 | 4320 | 60 | 3 | 8 |
| Meng-Che Shih | BGT | 250 | 2.6 | 1.321480231 | 10 | 2.3 | -20 | 230 | 1840 | 50 | 2 | 8 |
|  | SE | 500 | 2.3 | 1.458314095 | 10 | 3.8 | -120 | 380 | 3040 | 50 | 2 | 8 |
| Rocco Salvatore Calabrò et al | SE | 250 | 5 | 1.509966887 | 25 | 2.5 | 62.5 | 312.5 | 2500 | 25 | 5 | 8 |
|  | AE | 250 | 4 | 1.708800749 | 25 | 2.3 | 37.5 | 287.5 | 2300 | 25 | 5 | 8 |
| Alessandro Carvalho | RT | 250 | 2.2 | 1.768827295 | 8 | 3.5 | 30 | 280 | 3360 | 40 | 2 | 12 |
|  | BGT | 250 | 0 | 1.052510227 | 9 | 2.3 | -66 | 184 | 2208 | 40 | 2 | 12 |
|  | AE | 250 | 1.4 | 1.820439507 | 5 | 3.5 | 30 | 280 | 3360 | 40 | 2 | 12 |
| Ilaria Arcolin | AE | 750 | 3.3 | 0.975073962 | 13 | 2.3 | -60 | 690 | 2070 | 60 | 5 | 3 |
|  | AE | 1750 | 2.3 | 0.900347155 | 16 | 6 | 50 | 1800 | 5400 | 60 | 5 | 3 |
| Tuğba ATAN et al. | AE | 250 | 5.1 | 2.277498628 | 10 | 2.3 | 95 | 345 | 2070 | 30 | 5 | 6 |
|  | AE | 250 | 9.0 | 2.091650066 | 10 | 2 | 50 | 300 | 1800 | 30 | 5 | 6 |
|  | AE | 250 | 5.1 | 2.277498628 | 10 | 2.3 | 95 | 345 | 2070 | 30 | 5 | 6 |
|  | AE | 250 | 19.5 | 2.085905079 | 10 | 2 | 50 | 300 | 1800 | 30 | 5 | 6 |
| Dae-Hyouk Bang | AE | 750 | 2.7 | 1.336491676 | 10 | 2.3 | -60 | 690 | 2760 | 60 | 5 | 4 |
|  | AE | 1500 | 5.9 | 1.246647504 | 10 | 4.8 | -60 | 1440 | 5760 | 60 | 5 | 4 |
| Ilaria Carpinella et al | CON | 0 | 1.7 | 2.437314095 | 20 | 0 | 0 | 0 | 0 | 0 | 0 | 0 |
|  | SE | 250 | 4 | 1.989235739 | 17 | 2.3 | 60.5 | 310.5 | 2173.5 | 45 | 3 | 7 |
| Kim Chivers Seymour et al | CON | 0 | 0.2 | 0.430639335 | 211 | 0 | 0 | 0 | 0 | 0 | 0 | 0 |
|  | Mul_C | 1000 | 1.1 | 0.428939472 | 183 | 4.75 | -2.5 | 997.5 | 25935 | 30 | 7 | 26 |
| Silvia Rios Romenets et al. | CON | 0 | -2.6 | 1.587660753 | 15 | 0 | 0 | 0 | 0 | 0 | 0 | 0 |
|  | Dance | 250 | 0.7 | 0.707106781 | 18 | 3 | 110 | 360 | 4320 | 60 | 2 | 12 |
| Lucia Cugusi et al. | CON | 0 | -3 | 2.337306142 | 10 | 0 | 0 | 0 | 0 | 0 | 0 | 0 |
|  | AE | 500 | 6.6 | 2.165280582 | 10 | 4.8 | 76 | 576 | 6912 | 60 | 2 | 12 |
| G.FrazziAEa et al. | SE | 750 | 7.3 | 1.356957381 | 30 | 3.5 | -15 | 735 | 2940 | 35 | 6 | 4 |
|  | AE | 750 | 7.6 | 0.96970786 | 30 | 3.5 | -15 | 735 | 2940 | 35 | 6 | 4 |
| Pieter Ginis et al. | BGT | 250 | -0.89 | 1.077156751 | 18 | 2.3 | -43 | 207 | 1242 | 30 | 3 | 6 |
|  | SE | 250 | 1.35 | 1.161277314 | 20 | 2.3 | -43 | 207 | 1242 | 30 | 3 | 6 |
| Victoria A Goodwin et al. | CON | 0 | 0.5 | 1.414269681 | 63 | 0 | 0 | 0 | 0 | 0 | 0 | 0 |
|  | Mul_C | 250 | 5 | 1.289745178 | 61 | 4.75 | 35 | 285 | 2850 | 60 | 1 | 10 |
| Madeleine E. Hackney et al. | RT | 500 | 1.7 | 0.901249133 | 10 | 3.5 | -80 | 420 | 5460 | 60 | 2 | 13 |
|  | Dance | 250 | 3.8 | 1 | 9 | 3 | 110 | 360 | 4680 | 60 | 2 | 13 |
| C.C. Harro et al. | SE | 250 | 2.1 | 0.986407624 | 10 | 3 | 20 | 270 | 1620 | 30 | 3 | 6 |
|  | AE | 250 | 1.6 | 1.519210321 | 10 | 3.5 | 65 | 315 | 1890 | 30 | 3 | 6 |
| Adriano Zanardi da Silva | CON | 0 | -2.45 | 1.698729472 | 11 | 0 | 0 | 0 | 0 | 0 | 0 | 0 |
|  | AQE | 750 | 5.39 | 1.105185828 | 14 | 5.3 | -114 | 636 | 6360 | 60 | 2 | 10 |
| József Tollára | CON | 0 | -0.3 | 1.677900474 | 24 | 0 | 0 | 0 | 0 | 0 | 0 | 0 |
|  | SE | 1259 | 3.2 | 1.12 | 25 | 3.8 | -119 | 1140 | 5700 | 60 | 5 | 5 |
|  | AE | 1000 | 5.1 | 1.892 | 25 | 3.5 | 50 | 1050 | 5250 | 60 | 5 | 5 |
| José Eduardo Pompeu et al. | BGT | 250 | 1.2 | 1.033198916 | 16 | 2.3 | 26 | 276 | 1932 | 60 | 2 | 7 |
|  | SE | 500 | 1.5 | 0.888467782 | 16 | 3.8 | -44 | 456 | 3192 | 60 | 2 | 7 |
| Irene S.K. Wong-Yu et al. | CON | 0 | 0.8 | 1.273094353 | 39 | 0 | 0 | 0 | 0 | 0 | 0 | 0 |
|  | Mul_C | 500 | 10.7 | 1.148063056 | 41 | 4.75 | 70 | 570 | 4560 | 120 | 1 | 8 |
| Daniele Volpe et al. | BGT | 500 | 5.5 | 1.207683733 | 20 | 2.3 | -40 | 460 | 3680 | 40 | 5 | 8 |
|  | SE | 500 | 8.5 | 1.405702671 | 20 | 2.3 | -40 | 460 | 3680 | 40 | 5 | 8 |
| Zahra Kadivar | BGT | 500 | 5.8 | 0.318198052 | 8 | 2.5 | -125 | 375 | 2250 | 50 | 3 | 6 |
|  | SE | 250 | 6.56 | 0.430116263 | 8 | 2.3 | 95 | 345 | 2070 | 50 | 3 | 6 |
| Bakhshayesh Babak | BGT | 500 | 0.3 | 2.659448564 | 15 | 2.3 | -86 | 414 | 3312 | 60 | 3 | 8 |
|  | MBE | 500 | 10.7 | 4.510734604 | 15 | 3 | 40 | 540 | 4320 | 60 | 3 | 8 |
| Paolo Solla | CON | 0 | -0.7 | 1.368554411 | 17 | 0 | 0 | 0 | 0 | 0 | 0 | 0 |
|  | Dance | 1000 | 6.9 | 0.887764045 | 16 | 5.4 | -28 | 972 | 11664 | 90 | 2 | 12 |
| Kristi Michels | CON | 0 | 5.25 | 5.165762286 | 4 | 0 | 0 | 0 | 0 | 0 | 0 | 0 |
|  | Dance | 250 | 2.55 | 0.770483254 | 9 | 3 | 110 | 360 | 3600 | 60 | 2 | 10 |
| D Kunkel | CON | 0 | -1.7 | 1.846799033 | 15 | 0 | 0 | 0 | 0 | 0 | 0 | 0 |
|  | Dance | 750 | -0.7 | 1.111755369 | 30 | 5.4 | -102 | 648 | 6480 | 60 | 2 | 10 |
| Nicola Smania | CON | 0 | -0.8 | 1.649579071 | 27 | 3 | -50 | 450 | 3150 | 50 | 3 | 7 |
|  | SE | 500 | 5.3 | 1.09658561 | 28 | 3 | -50 | 450 | 3150 | 50 | 3 | 7 |
| Merrill R Landers | CON | 0 | -0.3 | 2.071472906 | 10 | 0 | 0 | 0 | 0 | 45 | 3 | 4 |
|  | BGT | 250 | 1.8 | 2.206127829 | 10 | 2.3 | 60.5 | 310.5 | 1242 | 45 | 3 | 4 |
|  | CON | 0 | -0.3 | 2.071472906 | 10 | 0 | 0 | 0 | 0 | 0 | 0 | 0 |
|  | SE | 250 | 3.3 | 2.206601665 | 11 | 2.3 | 60.5 | 310.5 | 1242 | 45 | 3 | 4 |
|  | BGT | 250 | 1.8 | 2.206127829 | 10 | 2.3 | 60.5 | 310.5 | 1242 | 45 | 3 | 4 |
|  | SE | 250 | 3.3 | 2.206601665 | 11 | 2.3 | 60.5 | 310.5 | 1242 | 45 | 3 | 4 |
|  | SE | 250 | 2.9 | 2.526657872 | 10 | 2.3 | 60.5 | 310.5 | 1242 | 45 | 3 | 4 |
| Hanan Khalil et al | CON | 0 | 1.5 | 2.066837725 | 11 | 0 | 0 | 0 | 0 | 0 | 0 | 0 |
|  | Mul_C | 750 | 1.9 | 1.433178286 | 15 | 4.75 | -108.75 | 641.25 | 5130 | 45 | 3 | 8 |
| Washington University School of Medicine | CON | 0 | 1 | 0.619978287 | 26 | 3 | 110 | 360 | 4320 | 60 | 2 | 12 |
|  | AE | 250 | 2 | 0.797447541 | 31 | 2.3 | 26 | 276 | 3312 | 60 | 2 | 12 |
|  | Dance | 250 | 2 | 0.583519384 | 39 | 3 | 110 | 360 | 4320 | 60 | 2 | 12 |
| Emine Eda Kurt et al | Mul_C | 1500 | 1 | 0.952102936 | 20 | 4.75 | -75 | 1425 | 7125 | 60 | 5 | 5 |
|  | AQE | 1500 | 6 | 3.067327827 | 20 | 5.3 | 90 | 1590 | 7950 | 60 | 5 | 5 |
| Leon CP Leal et al | CON | 0 | -3.22 | 1.628908782 | 27 | 0 | 0 | 0 | 0 | 0 | 0 | 0 |
|  | RT | 250 | 1.74 | 1.856494536 | 27 | 3.5 | -22.5 | 227.5 | 5460 | 32.5 | 2 | 24 |
| Hwa-Jin Lee, PhD et al. | CON | 0 | -0.4 | 0.837779804 | 16 | 0 | 0 | 0 | 0 | 0 | 0 | 0 |
|  | MBE | 250 | 0.9 | 0.490306027 | 25 | 3 | 110 | 360 | 2880 | 60 | 2 | 8 |
| Araceli Ortiz-Rubio et al | CON | 0 | 0.1 | 0.646394684 | 23 | 0 | 0 | 0 | 0 | 0 | 0 | 0 |
|  | RT | 500 | 2.3 | 0.750651891 | 23 | 3.5 | -80 | 420 | 3360 | 60 | 2 | 8 |
| Grazia Palamara et al. | Mul_C | 1250 | 7.3 | 0.949612924 | 17 | 4.75 | -110 | 1140 | 4560 | 60 | 4 | 4 |
|  | AQE | 1250 | 7.8 | 1.505480185 | 17 | 5.3 | 22 | 1272 | 5088 | 60 | 4 | 4 |
| A. Park etal | CON | 0 | 1.06 | 0.686326271 | 16 | 0 | 0 | 0 | 0 | 0 | 0 | 0 |
|  | RT | 750 | 1.31 | 0.789953585 | 15 | 3.5 | -120 | 630 | 30240 | 60 | 3 | 48 |
| Carla Silva-Batista et al. | CON | 0 | -4 | 3.716801768 | 13 | 0 | 0 | 0 | 0 | 0 | 0 | 0 |
|  | RT | 500 | 2.9 | 3.692403845 | 13 | 3.5 | -80 | 420 | 5040 | 60 | 2 | 12 |
| Carla Silva-Batista et al. | CON | 0 | -4 | 3.716801768 | 13 | 0 | 0 | 0 | 0 | 0 | 0 | 0 |
|  | Mul_C | 500 | 18.2 | 4.179344814 | 13 | 4.75 | 70 | 570 | 6840 | 60 | 2 | 12 |
| Maarten R.C. van den Heuvel et al. | SE | 250 | -1 | 1.53887134 | 16 | 2.3 | 26 | 276 | 1380 | 60 | 2 | 5 |
|  | SE | 500 | 1 | 0.901306242 | 17 | 3.8 | -44 | 456 | 2280 | 60 | 2 | 5 |
| Daniele Volpe1 et al. | BGT | 750 | 6 | 1.349727641 | 17 | 2.3 | -60 | 690 | 5520 | 60 | 5 | 8 |
|  | AQE | 1500 | 9.9 | 1.239781764 | 17 | 5.3 | 90 | 1590 | 12720 | 60 | 5 | 8 |
| Daniele Volpe1 et al. | Mul_C | 1500 | 6.9 | 2.234237718 | 11 | 4.75 | -75 | 1425 | 11400 | 60 | 5 | 8 |
|  | AQE | 1500 | 3.5 | 1.625754263 | 13 | 5.3 | 90 | 1590 | 12720 | 60 | 5 | 8 |
| Irene Cabrera-Martos et al. | CON | 0 | 0.39 | 0.834682847 | 22 | 3 | -95 | 405 | 3240 | 45 | 3 | 8 |
|  | RT | 500 | 2.75 | 0.71055676 | 22 | 3.5 | -27.5 | 472.5 | 3780 | 45 | 3 | 8 |
| José Ma Cancela | Mul_C | 750 | 0.07 | 0.44946635 | 5 | 4.75 | -37.5 | 712.5 | 5700 | 50 | 3 | 8 |
|  | AQE | 750 | 0.61 | 0.387427413 | 7 | 5.3 | 45 | 795 | 6360 | 50 | 3 | 8 |
| Tamine T.C. Capato et al. | CON | 0 | 0.1 | 1.149372111 | 48 | 0 | 0 | 0 | 0 | 0 | 0 | 0 |
|  | BGT | 250 | 1 | 1.041149365 | 50 | 2.3 | -43 | 207 | 1035 | 45 | 2 | 5 |
|  | SE | 250 | 7.1 | 0.800583493 | 56 | 3 | 20 | 270 | 1350 | 45 | 2 | 5 |
| Tamine T.C. Capato et al. | BGT | 250 | 1.5 | 2.326180704 | 18 | 2.3 | -43 | 207 | 0 | 45 | 2 |  |
|  | SE | 250 | 8.7 | 2.055464739 | 17 | 3 | 20 | 270 | 1350 | 45 | 2 | 5 |
| Qiang Gao et al. | CON | 0 | 0.39 | 1.465175232 | 39 | 0 | 0 | 0 | 0 | 0 | 0 | 5 |
|  | MBE | 500 | 4.16 | 1.581046523 | 37 | 3 | 40 | 540 | 6480 | 60 | 3 | 12 |
| Hanna Johansson et al. | CON | 0 | 0 | 0.816496581 | 6 | 0 | 0 | 0 | 0 | 0 | 0 | 0 |
|  | SE | 500 | 0 | 0.283473355 | 7 | 4.3 | 16 | 516 | 5160 | 60 | 2 | 10 |
| Conran Joseph et al. | CON | 0 | 0.74 | 0.537220858 | 48 | 0 | 0 | 0 | 0 | 0 | 0 | 0 |
|  | SE | 500 | 2.78 | 0.432273969 | 51 | 3 | 40 | 540 | 5400 | 60 | 3 | 10 |
| L. A. King al. | Mul_C | 1000 | 3.2 | 1.217374223 | 20 | 3 | -100 | 900 | 3600 | 75 | 4 | 4 |
|  | AE | 750 | 1.6 | 1.2489996 | 19 | 2.3 | -60 | 690 | 2760 | 75 | 4 | 4 |
| Se Hee Jung al. | CON | 0 | 0.26 | 0.310149733 | 42 | 0 | 0 | 0 | 0 | 0 | 0 | 0 |
|  | Mul_C | 750 | 0.94 | 0.360306057 | 44 | 3 | -30 | 720 | 4320 | 80 | 3 | 6 |
| Christian Schlenstedt et al | RT | 500 | 2.1 | 1.402081786 | 12 | 3.5 | -80 | 420 | 2940 | 60 | 2 | 7 |
|  | BGT | 250 | 0 | 1.932938178 | 8 | 2.3 | 26 | 276 | 1932 | 60 | 2 | 7 |
| Esther M. J. Bekkers, PhD et al. | AE | 250 | 0.99 | 0.195283366 | 59 | 2.3 | 60.5 | 310.5 | 1863 | 45 | 3 | 6 |
|  | SE | 500 | 1.89 | 0.215900216 | 62 | 3.8 | 13 | 513 | 3078 | 45 | 3 | 6 |
| Hsin-Hsuan Liu, PT et al. | BGT | 250 | 2.79 | 1.282260838 | 14 | 2.3 | 26 | 276 | 2208 | 60 | 2 | 8 |
|  | CON | 0 | 0.72 | 1.380964984 | 14 | 0 | 0 | 0 | 0 | 0 | 0 | 0 |
| Zhenlan Li et al. | MBE | 250 | 4.47 | 1.397075517 | 20 | 3 | 110 | 360 | 4320 | 60 | 2 | 12 |
|  | CON | 0 | 0.2 | 1.285684643 | 20 | 2.3 | 26 | 276 | 3312 | 60 | 2 | 12 |

## **List of included studies**

1. Wallén, M.B., et al., *Long-term effects of highly challenging balance training in Parkinson's disease-a randomized controlled trial.* Clin Rehabil, 2018. **32**(11): p. 1520-1529.

2. Pohl, P., et al., *Group-based music intervention in Parkinson's disease - findings from a mixed-methods study.* Clin Rehabil, 2020. **34**(4): p. 533-544.

3. Myers, P.S., et al., *Yoga Improves Balance and Low-Back Pain, but Not Anxiety, in People with Parkinson's Disease.* Int J Yoga Therap, 2020. **30**(1): p. 41-48.

4. Santos, S.M., et al., *Balance versus resistance training on postural control in patients with Parkinson's disease: a randomized controlled trial.* Eur J Phys Rehabil Med, 2017. **53**(2): p. 173-183.

5. Capecci, M., et al., *Postural rehabilitation and Kinesio taping for axial postural disorders in Parkinson's disease.* Arch Phys Med Rehabil, 2014. **95**(6): p. 1067-75.

6. Cherup, N.P., et al., *Yoga Meditation Enhances Proprioception and Balance in Individuals Diagnosed With Parkinson's Disease.* Percept Mot Skills, 2021. **128**(1): p. 304-323.

7. Conradsson, D., et al., *The Effects of Highly Challenging Balance Training in Elderly With Parkinson's Disease: A Randomized Controlled Trial.* Neurorehabil Neural Repair, 2015. **29**(9): p. 827-36.

8. Gandolfi, M., et al., *Virtual Reality Telerehabilitation for Postural Instability in Parkinson's Disease: A Multicenter, Single-Blind, Randomized, Controlled Trial.* Biomed Res Int, 2017. **2017**: p. 7962826.

9. Hackney, M.E. and G.M. Earhart, *Tai Chi improves balance and mobility in people with Parkinson disease.* Gait Posture, 2008. **28**(3): p. 456-60.

10. Khuzema, A., A. Brammatha, and V. Arul Selvan, *Effect of home-based Tai Chi, Yoga or conventional balance exercise on functional balance and mobility among persons with idiopathic Parkinson's disease: An experimental study.* Hong Kong Physiother J, 2020. **40**(1): p. 39-49.

11. Ni, M., et al., *Comparative Effect of Power Training and High-Speed Yoga on Motor Function in Older Patients With Parkinson Disease.* Arch Phys Med Rehabil, 2016. **97**(3): p. 345-354.e15.

12. Schlenstedt, C., et al., *Resistance versus Balance Training to Improve Postural Control in Parkinson's Disease: A Randomized Rater Blinded Controlled Study.* PLoS One, 2015. **10**(10): p. e0140584.

13. Ribas, C.G., et al., *Effectiveness of exergaming in improving functional balance, fatigue and quality of life in Parkinson's disease: A pilot randomized controlled trial.* Parkinsonism Relat Disord, 2017. **38**: p. 13-18.

14. Santos, P., et al., *Efficacy of the Nintendo Wii combination with Conventional Exercises in the rehabilitation of individuals with Parkinson's disease: A randomized clinical trial.* NeuroRehabilitation, 2019. **45**(2): p. 255-263.

15. Steib, S., et al., *Perturbation During Treadmill Training Improves Dynamic Balance and Gait in Parkinson's Disease: A Single-Blind Randomized Controlled Pilot Trial.* Neurorehabil Neural Repair, 2017. **31**(8): p. 758-768.

16. Xiao, C.M. and Y.C. Zhuang, *Effect of health Baduanjin Qigong for mild to moderate Parkinson's disease.* Geriatr Gerontol Int, 2016. **16**(8): p. 911-9.

17. Xiao, C., Y. Zhuang, and Y. Kang, *Effect of Health Qigong Baduanjin on Fall Prevention in Individuals with Parkinson's Disease.* J Am Geriatr Soc, 2016. **64**(11): p. e227-e228.

18. Yang, W.C., et al., *Home-based virtual reality balance training and conventional balance training in Parkinson's disease: A randomized controlled trial.* J Formos Med Assoc, 2016. **115**(9): p. 734-43.

19. Zhang, T.Y., et al., *Effects of Tai Chi and Multimodal Exercise Training on Movement and Balance Function in Mild to Moderate Idiopathic Parkinson Disease.* Am J Phys Med Rehabil, 2015. **94**(10 Suppl 1): p. 921-9.

20. Daneshmandi, H., S. Sayyar, and B.J.Z.J.o.R.i.M.S. Bakhshayesh, *The Effect of a Selective Pilates Program on Functional Balance and Falling Risk in Patients with Parkinson’s Disease.* 2017. **19**: p. 0-0.

21. Shih, M.C., et al., *Effects of a balance-based exergaming intervention using the Kinect sensor on posture stability in individuals with Parkinson's disease: a single-blinded randomized controlled trial.* J Neuroeng Rehabil, 2016. **13**(1): p. 78.

22. Calabrò, R.S., et al., *Walking to your right music: a randomized controlled trial on the novel use of treadmill plus music in Parkinson's disease.* J Neuroeng Rehabil, 2019. **16**(1): p. 68.

23. Abraham, A., et al., *Dynamic Neuro-Cognitive Imagery Improves Mental Imagery Ability, Disease Severity, and Motor and Cognitive Functions in People with Parkinson's Disease.* Neural Plast, 2018. **2018**: p. 6168507.

24. Carvalho, A., et al., *Comparison of strength training, aerobic training, and additional physical therapy as supplementary treatments for Parkinson's disease: pilot study.* Clin Interv Aging, 2015. **10**: p. 183-91.

25. Arcolin, I., et al., *Intensive cycle ergometer training improves gait speed and endurance in patients with Parkinson’s disease: A comparison with treadmill training.* Restorative Neurology and Neuroscience, 2015. **34**(1): p. 125-138.

26. Atan, T., et al., *Effects of different percentages of body weight-supported treadmill training in Parkinson’s disease: a double-blind randomized controlled trial.* Turkish Journal of Medical Sciences, 2019. **49**(4): p. 999-1007.

27. Bang, D.-H. and W.-S. Shin, *Effects of an intensive Nordic walking intervention on the balance function and walking ability of individuals with Parkinson’s disease: a randomized controlled pilot trial.* Aging Clinical and Experimental Research, 2016. **29**(5): p. 993-999.

28. Carpinella, I., et al., *Wearable Sensor-Based Biofeedback Training for Balance and Gait in Parkinson Disease: A Pilot Randomized Controlled Trial.* Archives of Physical Medicine and Rehabilitation, 2017. **98**(4): p. 622-630.e3.

29. Chivers Seymour, K., et al., *Multicentre, randomised controlled trial of PDSAFE, a physiotherapist-delivered fall prevention programme for people with Parkinson’s.* Journal of Neurology, Neurosurgery & Psychiatry, 2019. **90**(7): p. 774-782.

30. Rios Romenets, S., et al., *Tango for treatment of motor and non-motor manifestations in Parkinson's disease: A randomized control study.* Complementary Therapies in Medicine, 2015. **23**(2): p. 175-184.

31. Cugusi, L., et al., *Effects of a Nordic Walking program on motor and non-motor symptoms, functional performance and body composition in patients with Parkinson’s disease.* NeuroRehabilitation, 2015. **37**(2): p. 245-254.

32. Frazzitta, G., et al., *Crossover versus Stabilometric Platform for the Treatment of Balance Dysfunction in Parkinson’s Disease: A Randomized Study.* BioMed Research International, 2015. **2015**: p. 1-7.

33. Ginis, P., et al., *Feasibility and effects of home-based smartphone-delivered automated feedback training for gait in people with Parkinson's disease: A pilot randomized controlled trial.* Parkinsonism & Related Disorders, 2016. **22**: p. 28-34.

34. Goodwin, V.A., et al., *An exercise intervention to prevent falls in people with Parkinson's disease: a pragmatic randomised controlled trial.* Journal of Neurology, Neurosurgery & Psychiatry, 2011. **82**(11): p. 1232-1238.

35. Hackney, M.E., et al., *Effects of Tango on Functional Mobility in Parkinson's Disease: A Preliminary Study.* Journal of Neurologic Physical Therapy, 2007. **31**(4): p. 173-179.

36. Harro, C.C., et al., *The effects of speed-dependent treadmill training and rhythmic auditory-cued overground walking on balance function, fall incidence, and quality of life in individuals with idiopathic Parkinson's disease: A randomized controlled trial.* NeuroRehabilitation, 2014. **34**(3): p. 541-556.

37. Silva, A.Z.d. and V.L. Israel, *Effects of dual-task aquatic exercises on functional mobility, balance and gait of individuals with Parkinson's disease: A randomized clinical trial with a 3-month follow-up.* Complementary Therapies in Medicine, 2019. **42**: p. 119-124.

38. Tollár, J., F. Nagy, and T. Hortobágyi, *Vastly Different Exercise Programs Similarly Improve Parkinsonian Symptoms: A Randomized Clinical Trial.* Gerontology, 2019. **65**(2): p. 120-127.

39. Pompeu, J.E., et al., *Effect of Nintendo Wii™-based motor and cognitive training on activities of daily living in patients with Parkinson's disease: A randomised clinical trial.* Physiotherapy, 2012. **98**(3): p. 196-204.

40. Wong-Yu, I.S.K. and M.K.Y. Mak, *Multi-dimensional balance training programme improves balance and gait performance in people with Parkinson's disease: A pragmatic randomized controlled trial with 12-month follow-up.* Parkinsonism & Related Disorders, 2015. **21**(6): p. 615-621.

41. Volpe, D., M.G. Giantin, and A. Fasano, *A wearable proprioceptive stabilizer (Equistasi®) for rehabilitation of postural instability in Parkinson's disease: a phase II randomized double-blind, double-dummy, controlled study.* PLoS One, 2014. **9**(11): p. e112065.

42. Kadivar, Z., et al., *Effect of Step Training and Rhythmic Auditory Stimulation on Functional Performance in Parkinson Patients.* Neurorehabilitation and Neural Repair, 2011. **25**(7): p. 626-635.

43. Bakhshayesh, B., S. Sayyar, and H. Daneshmandi, *Pilates Exercise and Functional Balance in Parkinson's Disease.* Caspian Journal of Neurological Sciences, 2017. **3**(8): p. 25-38.

44. Solla, P., et al., *Sardinian Folk Dance for Individuals with Parkinson's Disease: A Randomized Controlled Pilot Trial.* The Journal of Alternative and Complementary Medicine, 2019. **25**(3): p. 305-316.

45. Michels, K., et al., *“Dance Therapy” as a psychotherapeutic movement intervention in Parkinson’s disease.* Complementary Therapies in Medicine, 2018. **40**: p. 248-252.

46. Kunkel, D., et al., *A randomized controlled feasibility trial exploring partnered ballroom dancing for people with Parkinson’s disease.* Clinical Rehabilitation, 2017. **31**(10): p. 1340-1350.

47. Smania, N., et al., *Effect of Balance Training on Postural Instability in Patients With Idiopathic Parkinson’s Disease.* Neurorehabilitation and Neural Repair, 2010. **24**(9): p. 826-834.

48. Khalil, H., et al., *A pilot study of a minimally supervised home exercise and walking program for people with Parkinson's disease in Jordan.* Neurodegener Dis Manag, 2017. **7**(1): p. 73-84.

49. Landers, M.R., et al., *Does attentional focus during balance training in people with Parkinson’s disease affect outcome? A randomised controlled clinical trial.* Clinical Rehabilitation, 2015. **30**(1): p. 53-63.

50. Kurt, E.E., et al., *Effects of Ai Chi on balance, quality of life, functional mobility, and motor impairment in patients with Parkinson’s disease.* Disability and Rehabilitation, 2017. **40**(7): p. 791-797.

51. Leal, L.C.P., et al., *Low‐volume resistance training improves the functional capacity of older individuals with Parkinson's disease.* Geriatrics & Gerontology International, 2019. **19**(7): p. 635-640.

52. Lee, H.-J., et al., *Turo (Qi Dance) Program for Parkinson’s Disease Patients: Randomized, Assessor Blind, Waiting-List Control, Partial Crossover Study.* Explore, 2018. **14**(3): p. 216-223.

53. Ortiz-Rubio, A., et al., *Effects of a resistance training program on balance and fatigue perception in patients with Parkinson's disease: A randomized controlled trial.* Medicina Clínica, 2018. **150**(12): p. 460-464.

54. Palamara, G., et al., *Land Plus Aquatic Therapy Versus Land-Based Rehabilitation Alone for the Treatment of Balance Dysfunction in Parkinson Disease: A Randomized Controlled Study With 6-Month Follow-Up.* Archives of Physical Medicine and Rehabilitation, 2017. **98**(6): p. 1077-1085.

55. Park, A., et al., *Effects of a formal exercise program on Parkinson's disease: A pilot study using a delayed start design.* Parkinsonism & Related Disorders, 2014. **20**(1): p. 106-111.

56. Silva-Batista, C., et al., *Balance and fear of falling in subjects with Parkinson’s disease is improved after exercises with motor complexity.* Gait & Posture, 2018. **61**: p. 90-97.

57. van den Heuvel, M.R., et al., *Effects of augmented visual feedback during balance training in Parkinson's disease: a pilot randomized clinical trial.* Parkinsonism Relat Disord, 2014. **20**(12): p. 1352-8.

58. Volpe, D., et al., *Comparing the effects of hydrotherapy and land-based therapy on balance in patients with Parkinson’s disease: a randomized controlled pilot study.* Clinical Rehabilitation, 2014. **28**(12): p. 1210-1217.

59. Volpe, D., et al., *Water-based vs. non-water-based physiotherapy for rehabilitation of postural deformities in Parkinson’s disease: a randomized controlled pilot study.* Clinical Rehabilitation, 2016. **31**(8): p. 1107-1115.

60. Cabrera-Martos, I., et al., *Effects of a core stabilization training program on balance ability in persons with Parkinson's disease: a randomized controlled trial.* Clin Rehabil, 2020. **34**(6): p. 764-772.

61. Cancela, J.M., et al., *Effects of a High-Intensity Progressive-Cycle Program on Quality of Life and Motor Symptomatology in a Parkinson's Disease Population: A Pilot Randomized Controlled Trial.* Rejuvenation Research, 2020. **23**(6): p. 508-515.

62. Capato, T.T.C., et al., *Multimodal Balance Training Supported by Rhythmical Auditory Stimuli in Parkinson’s Disease: A Randomized Clinical Trial.* Journal of Parkinson's Disease, 2020. **10**(1): p. 333-346.

63. Capato, T.T.C., et al., *Effects of multimodal balance training supported by rhythmical auditory stimuli in people with advanced stages of Parkinson's disease: a pilot randomized clinical trial.* Journal of the Neurological Sciences, 2020. **418**.

64. Gao, Q., et al., *Effects of Tai Chi on balance and fall prevention in Parkinson’s disease: a randomized controlled trial.* Clinical Rehabilitation, 2014. **28**(8): p. 748-753.

65. Johansson, H., et al., *Feasibility Aspects of Exploring Exercise-Induced Neuroplasticity in Parkinson’s Disease: A Pilot Randomized Controlled Trial.* Parkinson's Disease, 2020. **2020**: p. 1-12.

66. Joseph, C., et al., *Cost-effectiveness of the HiBalance training program for elderly with Parkinson’s disease: analysis of data from a randomized controlled trial.* Clinical Rehabilitation, 2018. **33**(2): p. 222-232.

67. King, L.A., et al., *Exploring Outcome Measures for Exercise Intervention in People with Parkinson’s Disease.* Parkinson's Disease, 2013. **2013**: p. 1-9.

68. Jung, S.H., et al., *Effects of the agility boot camp with cognitive challenge (ABC-C) exercise program for Parkinson’s disease.* npj Parkinson's Disease, 2020. **6**(1).

69. Schlenstedt, C., et al., *Moderate Frequency Resistance and Balance Training Do Not Improve Freezing of Gait in Parkinson's Disease: A Pilot Study.* Frontiers in Neurology, 2018. **9**.

70. Bekkers, E.M.J., et al., *Do Patients With Parkinson’s Disease With Freezing of Gait Respond Differently Than Those Without to Treadmill Training Augmented by Virtual Reality?* Neurorehabilitation and Neural Repair, 2020. **34**(5): p. 440-449.

71. Liu, H.-H., et al., *Balance Training Modulates Cortical Inhibition in Individuals with Parkinson’s Disease: A Randomized Controlled Trial.* Neurorehabilitation and Neural Repair, 2022. **36**(9): p. 613-620.

72. Li, Z., et al., *Comparison of Wuqinxi Qigong with Stretching on Single- and Dual-Task Gait, Motor Symptoms and Quality of Life in Parkinson’s Disease: A Preliminary Randomized Control Study.* International Journal of Environmental Research and Public Health, 2022. **19**(13).

73.  *Washington University School of Medicine. Exercise and Parkinson's: Comparing Interventions and Exploring Neural Mechanisms. 2018.*

# **Supplementary File 4: Three key assumptions of network meta-analysis in our study**

## (1) Consistency Test

To check for consistency at the treatment level using UME, we fitted direct relative effects between each group and study in the NMA studies modeled only without assuming a consistency relationship, concerning treatment (Dias et al., 2013). If the consistency assumption holds, then the results of UME-established modeling and NMA would be very similar. size (Higgins et al., 2012). We compared the consistency of these models by examining the bias, the number of estimated parameters in the network, and the Deviance Information Criterion (DIC) metric. The results indicate a good fit, as these parameters show good agreement between the models (Supplementary Table 4.1). In addition to visually verifying the consistency of the models using the residuals, scatterplot shapes were plotted to investigate the fit of the different data points by comparing the different models through scatterplots (Supplementary 4 Table 4.1; Supplementary Figure 3).

### **Table 4.1. Consistent and UME models fit the comparison**

**
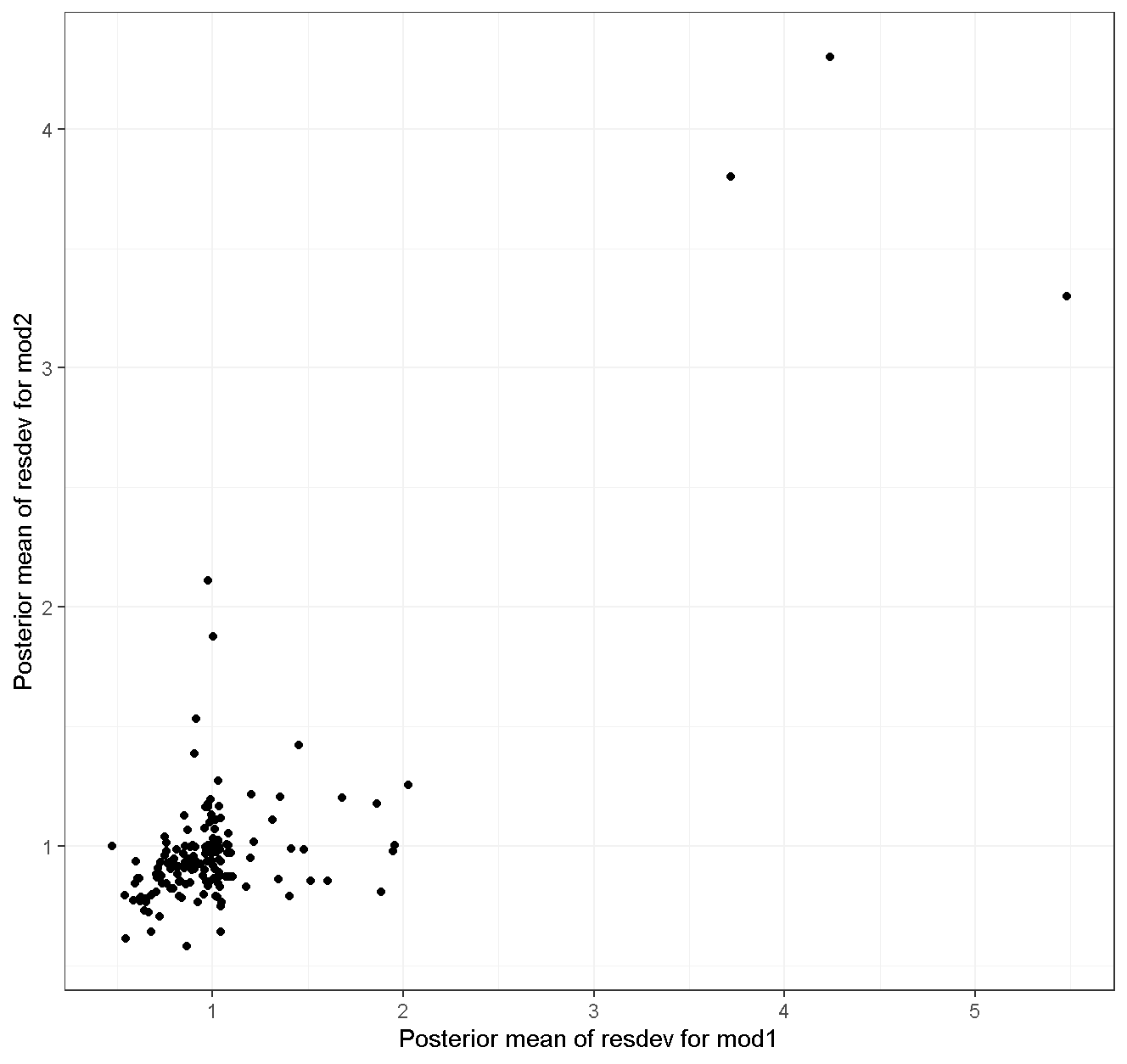
**PD: Number of estimated parameters; DIC: Deviance Informative Criterion; SD: Standard Deviation; UME: Unrelated Mean Effects. Scientific literature indicated that the main indicator to assess the model fit is the DIC. As lower DIC, better fit

### **Figure 4.1:** Validation Model Consistency Scatterplot

| **Model** | **pD** | **Residual deviance** | **Deviance** | **DIC** | **SD** |
| --- | --- | --- | --- | --- | --- |
| Consistent | 128.5 | 164.505 | 517.330 | 645.5 | 0.237 |
| UME | 134 | 161.420 | 514.245 | 647.6 | 0.411 |

## **(2) Transitivity Test**

Transitivity NMA is based on the assumption of potential indirect/mixed comparisons, which implies that direct and indirect evidence are consistent in their estimates of treatment effects, but with the usual variability that occurs in meta-analyses under random effects models (Shim et al., 2017). This assumption is equivalent to heterogeneity in "standard" meta-analyses. Following previous recommendations, transmissibility was assessed at a deeper level of the network (i.e., the treatment level).

We assessed transitivity through the MBNMA node splitting method. The results showed that there was no inconsistency in our study (p>0.05), we used the MBNMA node splitting method to assess inconsistency. Similar effects indicate good agreement. Supplementary 4 Table 2 and Figure 4 (density plot) give the results of the node-split analysis of inconsistency and transferability in this meta-analysis. The results show that there is no inconsistency in our study (p>0.05). The method categorizes the contribution of a given treatment comparison into direct and indirect evidence. The similar effects indicate good and materiality.

### Table 4.2: Node-splitting analysis of inconsistency

| **Comparison** | **p-value** | **Median** | **2.50%** | **97.50%** |
| --- | --- | --- | --- | --- |
| SE_500 vs SE_250 | 0.108 |  |  |  |
| -> direct |  | 1.996 | -3.015 | 7.103 |
| -> indirect |  | 0.228 | 0.014 | 0.664 |
| -> MBNMA |  | 0.242 | 0.011 | 0.698 |
|  |  |  |  |  |
| SE_250 vs RT_250 | 0.573 |  |  |  |
| -> direct |  | 0.259 | -3.661 | 4.223 |
| -> indirect |  | -0.736 | -2.486 | 1.056 |
| -> MBNMA |  | -0.657 | -2.295 | 0.887 |
|  |  |  |  |  |
| MulC_500 vs MBE_250 | 0.728 |  |  |  |
| -> direct |  | 0.542 | -3.691 | 4.717 |
| -> indirect |  | 0.977 | -1.365 | 3.412 |
| -> MBNMA |  | 0.867 | -1.114 | 2.86 |
|  |  |  |  |  |
| RT_500 vs Dance_250 | 0.439 |  |  |  |
| -> direct |  | -2.075 | -6.777 | 2.404 |
| -> indirect |  | 0.547 | -2.099 | 3.172 |
| -> MBNMA |  | 0.861 | -1.39 | 3.216 |
|  |  |  |  |  |
| SE_750 vs BGT_750 | 0.18 |  |  |  |
| -> direct |  | 3.054 | -2.246 | 8.402 |
| -> indirect |  | -1.232 | -2.727 | 0.34 |
| -> MBNMA |  | -1.336 | -2.742 | 0.112 |
|  |  |  |  |  |
| SE_750 vs BGT_500 | 0.448 |  |  |  |
| -> direct |  | -0.45 | -5.012 | 4.211 |
| -> indirect |  | -1.601 | -3.034 | -0.148 |
| -> MBNMA |  | -1.421 | -2.786 | -0.038 |
|  |  |  |  |  |
| SE_250 vs BGT_500 | 0.394 |  |  |  |
| -> direct |  | 0.746 | -3.137 | 4.533 |
| -> indirect |  | -1.069 | -2.533 | 0.34 |
| -> MBNMA |  | -1.065 | -2.344 | 0.318 |
|  |  |  |  |  |
| RT_500 vs BGT_500 | 0.367 |  |  |  |
| -> direct |  | -3.883 | -9.598 | 1.599 |
| -> indirect |  | -1.21 | -3.049 | 0.794 |
| -> MBNMA |  | -0.64 | -2.472 | 1.179 |
|  |  |  |  |  |
| SE_750 vs BGT_250 | 0.23 |  |  |  |
| -> direct |  | 1.775 | -4.771 | 8.225 |
| -> indirect |  | -1.624 | -2.968 | -0.333 |
| -> MBNMA |  | -1.621 | -2.909 | -0.381 |
|  |  |  |  |  |
| SE_500 vs BGT_250 | 0.332 |  |  |  |
| -> direct |  | 0.095 | -2.786 | 3.003 |
| -> indirect |  | -1.857 | -3.271 | -0.541 |
| -> MBNMA |  | -1.524 | -2.71 | -0.333 |
|  |  |  |  |  |
| SE_250 vs BGT_250 | 0.015 |  |  |  |
| -> direct |  | 3.99 | 1.724 | 6.286 |
| -> indirect |  | -0.468 | -1.78 | 0.767 |
| -> MBNMA |  | -1.262 | -2.414 | -0.03 |
|  |  |  |  |  |
| RT_500 vs BGT_250 | 0.352 |  |  |  |
| -> direct |  | 2.046 | -1.902 | 5.802 |
| -> indirect |  | -0.552 | -2.484 | 1.404 |
| -> MBNMA |  | -0.827 | -2.581 | 0.88 |
|  |  |  |  |  |
| MBE_250 vs BGT_250 | 0.285 |  |  |  |
| -> direct |  | 1.675 | -2.619 | 6.044 |
| -> indirect |  | -1.509 | -3.533 | 0.466 |
| -> MBNMA |  | -1.47 | -3.323 | 0.293 |
|  |  |  |  |  |
| MulC_1200 vs AQE_1200 | 0.659 |  |  |  |
| -> direct |  | -0.655 | -5.761 | 4.403 |
| -> indirect |  | -1.496 | -3.974 | 1.13 |
| -> MBNMA |  | -1.255 | -3.538 | 1.058 |
|  |  |  |  |  |
| MulC_1000 vs AQE_1200 | 0.326 |  |  |  |
| -> direct |  | -4.979 | -12.267 | 2.281 |
| -> indirect |  | -0.916 | -3.286 | 1.612 |
| -> MBNMA |  | -1.298 | -3.578 | 1.004 |
|  |  |  |  |  |
| BGT_500 vs AQE_1000 | 0.082 |  |  |  |
| -> direct |  | -3.992 | -9.183 | 1.365 |
| -> indirect |  | 3.553 | 0.508 | 6.834 |
| -> MBNMA |  | 3.641 | 1.027 | 6.362 |
|  |  |  |  |  |
| MulC_750 vs AQE_750 | 0.722 |  |  |  |
| -> direct |  | -0.606 | -4.534 | 3.406 |
| -> indirect |  | -1.569 | -4.2 | 1.146 |
| -> MBNMA |  | -1.17 | -3.333 | 1.029 |
|  |  |  |  |  |
| MulC_750 vs AQE_500 | 0.23 |  |  |  |
| -> direct |  | 3.549 | -2.972 | 9.903 |
| -> indirect |  | -1.449 | -3.647 | 0.857 |
| -> MBNMA |  | -0.948 | -3.07 | 1.211 |
|  |  |  |  |  |
| MulC_1000 vs AE_750 | 0.654 |  |  |  |
| -> direct |  | 1.549 | -3.45 | 6.647 |
| -> indirect |  | 0.28 | -2.615 | 3.195 |
| -> MBNMA |  | 0.547 | -1.939 | 3.053 |
|  |  |  |  |  |
| AE_1200 vs AE_500 | 0.121 |  |  |  |
| -> direct |  | 3.204 | -2.115 | 8.234 |
| -> indirect |  | 0.366 | 0.029 | 1.061 |
| -> MBNMA |  | 0.397 | 0.028 | 1.101 |
|  |  |  |  |  |
| AE_750 vs AE_500 | 0.054 |  |  |  |
| -> direct |  | 3.784 | -3.525 | 10.862 |
| -> indirect |  | 0.203 | 0.015 | 0.555 |
| -> MBNMA |  | 0.214 | 0.016 | 0.57 |
|  |  |  |  |  |
| SE_500 vs AE_250 | 0.551 |  |  |  |
| -> direct |  | 0.935 | -2.749 | 4.774 |
| -> indirect |  | -0.065 | -1.624 | 1.528 |
| -> MBNMA |  | -0.225 | -1.687 | 1.308 |
|  |  |  |  |  |
| SE_250 vs AE_250 | 0.76 |  |  |  |
| -> direct |  | 0.849 | -1.98 | 3.514 |
| -> indirect |  | 0.419 | -1.356 | 2.28 |
| -> MBNMA |  | 0.032 | -1.391 | 1.577 |
|  |  |  |  |  |
| RT_250 vs AE_250 | 0.55 |  |  |  |
| -> direct |  | 0.7 | -4.996 | 6.468 |
| -> indirect |  | 0.855 | -1.127 | 2.916 |
| -> MBNMA |  | 0.712 | -1.153 | 2.71 |
|  |  |  |  |  |
| BGT_250 vs AE_250 | 0.341 |  |  |  |
| -> direct |  | -1.554 | -7.16 | 3.995 |
| -> indirect |  | 1.256 | -0.5 | 3.203 |
| -> MBNMA |  | 1.293 | -0.459 | 3.071 |
|  |  |  |  |  |
| AE_500 vs AE_250 | 0.196 |  |  |  |
| -> direct |  | -0.896 | -5.66 | 3.75 |
| -> indirect |  | 0.519 | 0.049 | 1.196 |
| -> MBNMA |  | 0.499 | 0.047 | 1.135 |
|  |  |  |  |  |
| SE_1200 vs Placebo_0 | 0.458 |  |  |  |
| -> direct |  | 2.906 | -2.288 | 7.67 |
| -> indirect |  | 3.101 | 1.829 | 4.552 |
| -> MBNMA |  | 3.073 | 1.842 | 4.478 |
|  |  |  |  |  |
| SE_1000 vs Placebo_0 | 0.481 |  |  |  |
| -> direct |  | 2.064 | -2.019 | 6.309 |
| -> indirect |  | 3.128 | 1.782 | 4.62 |
| -> MBNMA |  | 3.043 | 1.83 | 4.412 |
|  |  |  |  |  |
| RT_750 vs Placebo_0 | 0.383 |  |  |  |
| -> direct |  | 0.232 | -3.988 | 4.434 |
| -> indirect |  | 2.66 | 0.909 | 4.511 |
| -> MBNMA |  | 2.299 | 0.755 | 3.941 |
|  |  |  |  |  |
| MulC_250 vs Placebo_0 | 0.456 |  |  |  |
| -> direct |  | 4.515 | -0.972 | 9.679 |
| -> indirect |  | 3.238 | 1.719 | 5.084 |
| -> MBNMA |  | 3.36 | 1.872 | 5.064 |
|  |  |  |  |  |
| MBE_750 vs Placebo_0 | 0.286 |  |  |  |
| -> direct |  | 0.643 | -4.01 | 5.211 |
| -> indirect |  | 3.957 | 1.972 | 6.05 |
| -> MBNMA |  | 3.408 | 1.697 | 5.29 |
|  |  |  |  |  |
| Dance_1000 vs Placebo_0 | 0.194 |  |  |  |
| -> direct |  | 7.534 | 2.718 | 12.412 |
| -> indirect |  | 2.651 | 0.138 | 5.564 |
| -> MBNMA |  | 3.817 | 1.454 | 6.418 |
|  |  |  |  |  |
| Dance_750 vs Placebo_0 | 0.374 |  |  |  |
| -> direct |  | 0.868 | -4.721 | 6.52 |
| -> indirect |  | 4.352 | 1.579 | 7.156 |
| -> MBNMA |  | 3.722 | 1.423 | 6.171 |
|  |  |  |  |  |
| AE_1000 vs Placebo_0 | 0.457 |  |  |  |
| -> direct |  | 5.474 | -0.793 | 11.429 |
| -> indirect |  | 3.234 | 1.115 | 5.845 |
| -> MBNMA |  | 3.511 | 1.485 | 5.92 |
|  |  |  |  |  |


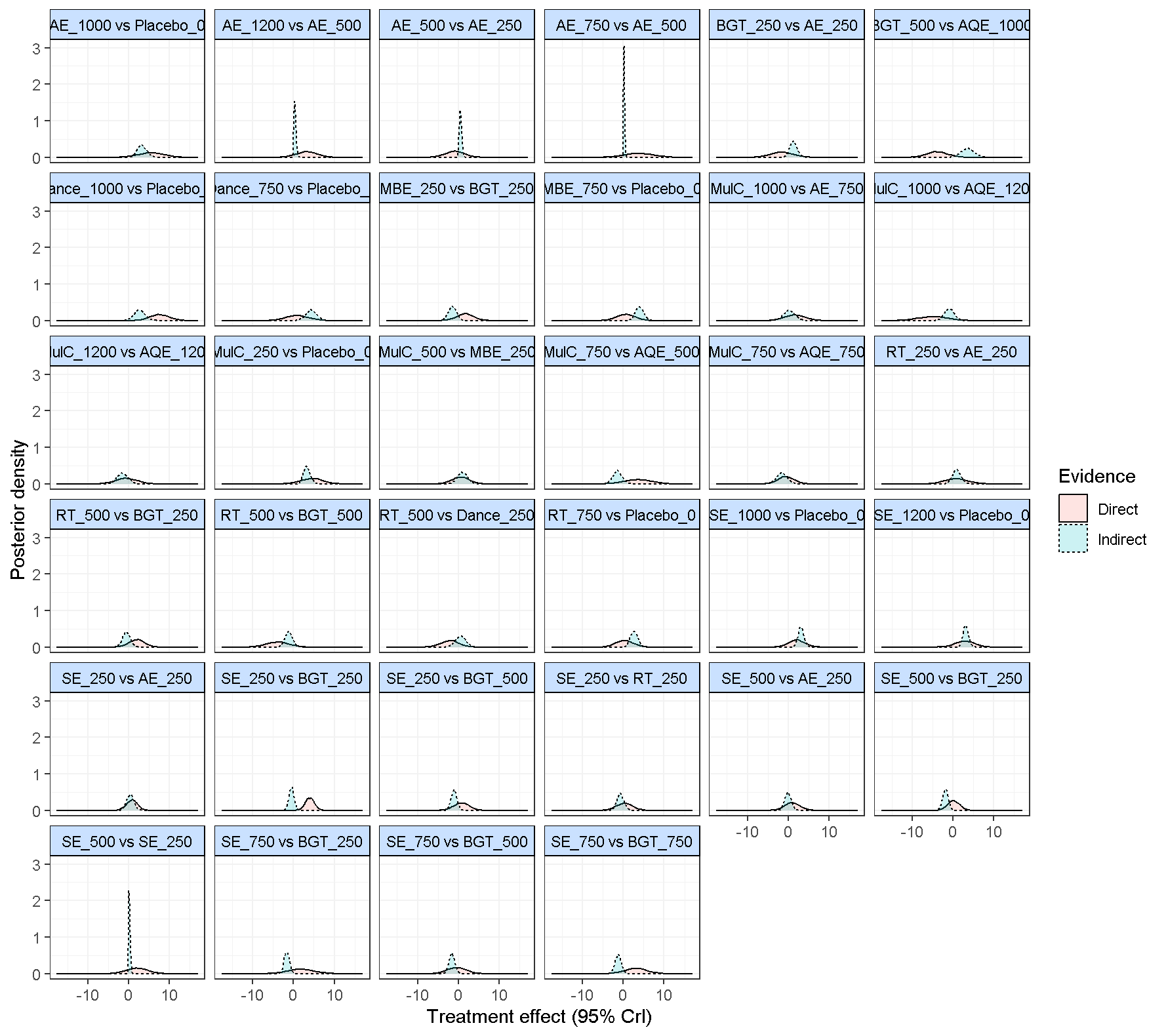


### **Figure 4.2:** Node splitting analysis (density plot)

# **Supplementary 5: The relationship of Non-linear model**

We investigated possible nonlinear associations by modeling exercise using restricted cubic spline strips located at three nodes in the 25th, 50th, 75th, and 100th percentiles of the distribution. We identified non-linear relationships across intervention modalities and different doses, which were subsequently used in a model-based network meta-analysis (MBNMA). Supplementary 5 Figure 1 shows the different responses of each dose to different types of exercise.


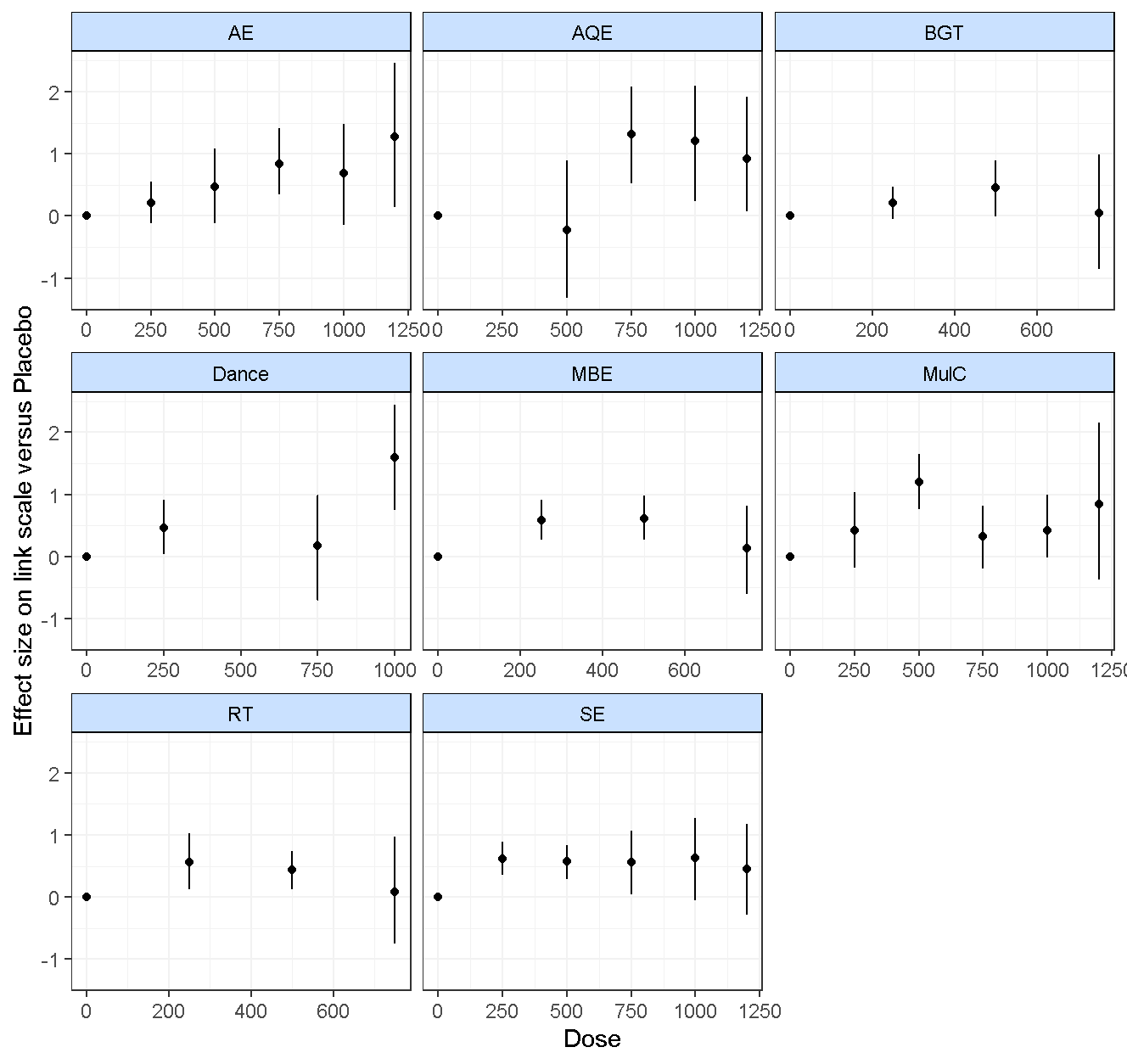


Figure 5.1: “Split” NMA of different exercise treatment agents. The values after the type of exercises correspond to the dose of that intervention AE: Aerobic exercise; Dance; AQE: Aquatic Exercise; MBE: Mind-body Exercise; Mul_C: Mixed Exercise Program; BGT: Balance and Gait Training; RT: Resistance Training; SE: Sensory Exercise; TT: Treadmill Training;

Table 5.1: the fit indices from each of the models fitted. For our data, restricted cubic splines show the best fit and were therefore used in subsequent analyses.

| **Model** | **DIC** | **SD** | **Deviance** | **Residual deviance** | **pD** |
| --- | --- | --- | --- | --- | --- |
| Emax  (common treatment effects) | 677.9 | NA | 592.773 | 239.948 | 85.8 |
| Emax  (random treatment effects) | 635.0 | 0.311 | 516.586 | 163.761 | 119.4 |
| Exponential  (common treatment effects) | 679.4 | NA | 595.249 | 242.425 | 84.7 |
| Exponential  (random treatment effects) | 638.4 | 0.311 | 518.673 | 165.849 | 120.7 |
| Restricted cubic spline  (common treatment effects; 3 knots) | 667.8 | NA | 575.602 | 222.777 | 92.9 |
| Restricted cubic spline  (random treatment effects; 3 knots) | 631.8 | 0.306 | 515.653 | 161.627 | 124.7 |
| Non-parametric monotonically up (common treatment effects) | 675.5 | NA | 583.868 | 231.043 | 92.4 |
| Non-parametric monotonically up (random treatment effects) | 636.2 | 0.361 | 510.047 | 157.222 | 126.7 |
| Linear (common treatment effects) | 714.0 | NA | 629.463 | 276.638 | 85.3 |
| Linear (random treatment effects) | 637.1 | 0.372 | 511.878 | 159.053 | 125.6 |

DIC = Deviance Information Criterion; SD = Between-study Standard Deviation; pD: Number of estimated parameters; NA = Not Applicable. The SD is presented as the main value and (95% Credible Intervals).

(2) In addition to identifying the model of restricted cubic spline strips, the bias plots showing the contribution of each data point to the residuals also help to confirm the robustness of the model choice.

The contribution of each data point to the deviation from the posterior mean is approximately 1, indicating a good model fit. The deviation plot for the treatment effect (Supplementary 5 Figure 2) confirms the robustness of our model choice.


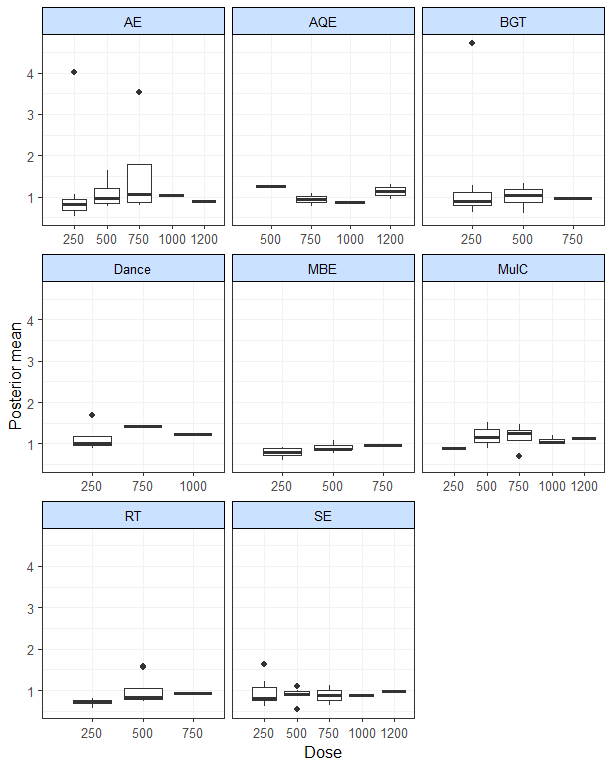


### **Figure 5.2**: Deviance box plot at different exercise levels

#
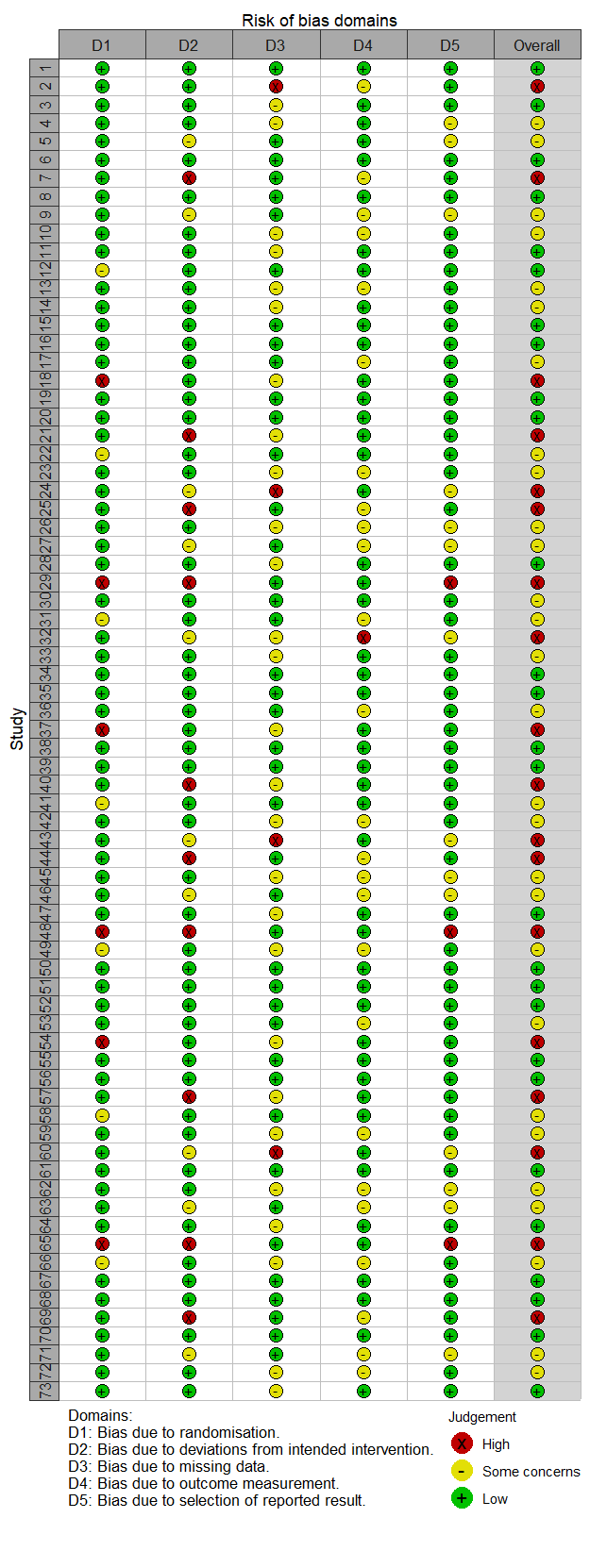
**Supplementary File 6:** **Study-level risk of bias analysis**

**Reference**

1. Dias, S., Sutton, A. J., Ades, A. E., & Welton, N. J. (2013). Evidence synthesis for decision making 2: a generalized linear modeling framework for pairwise and network meta-analysis of randomized controlled trials. Med Decis Making, 33(5), 607-617. https://doi.org/10.1177/0272989x12458724
2. Dias, S., Welton, N. J., Sutton, A. J., & Ades, A. E. (2013). Evidence synthesis for decision making 1: introduction. Med Decis Making, 33(5), 597-606. https://doi.org/10.1177/0272989x13487604
3. Higgins, J. P., Jackson, D., Barrett, J. K., Lu, G., Ades, A. E., & White, I. R. (2012). Consistency and inconsistency in network meta-analysis: concepts and models for multi-arm studies. Res Synth Methods, 3(2), 98-110. https://doi.org/10.1002/jrsm.1044
4. Rouse, B., Chaimani, A., & Li, T. (2017). Network meta-analysis: an introduction for clinicians. Intern Emerg Med, 12(1), 103-111. https://doi.org/10.1007/s11739-016-1583-7
5. Shim, S., Yoon, B. H., Shin, I. S., & Bae, J. M. (2017). Network meta-analysis: application and practice
using Stata. Epidemiol Health, 39, e2017047. https://doi.org/10.4178/epih.e2017047

# **Supplementary File 7: PRISMA Checklist**

| **Section/Topic** | **Item #** | **Checklist Item** | **Reported on Page #** |
| --- | --- | --- | --- |
| **TITLE** |  |  |  |
| Title | 1 | Identify the report as a systematic review *incorporating a network meta-analysis (or related form of meta-analysis).* | 1 |
| **ABSTRACT** |  |  |  |
| Structured summary | 2 | Provide a structured summary including, as applicable:  **Background:** main objectives  **Methods:** data sources; study eligibility criteria, participants, and interventions; study appraisal; and *synthesis methods, such as network meta-analysis.*  **Results:** number of studies and participants identified; summary estimates with corresponding confidence/credible intervals; *treatment rankings may also be discussed. Authors may choose to summarize pairwise comparisons against a chosen treatment included in their analyses for brevity.*  **Discussion/Conclusions:** limitations; conclusions and implications of findings.  **Other:** primary source of funding; systematic review registration number with registry name. | 2 |
| **INTRODUCTION** |  |  |  |
| Rationale | 3 | Describe the rationale for the review in the context of what is already known*, including mention of why a network meta-analysis has been conducted.* | 3-4 |
| Objectives | 4 | Provide an explicit statement of questions being addressed, concerning participants, interventions, comparisons, outcomes, and study design (PICOS). | 4 |
|  |  |  |  |
| **METHODS** |  |  |  |
| Protocol and registration | 5 | Indicate whether a review protocol exists and if and where it can be accessed (e.g., Web address); and, if available, provide registration information, including registration number. | 4  CRD 42024517241 |
| Eligibility criteria | 6 | Specify study characteristics (e.g., PICOS, length of follow-up) and report characteristics (e.g., years considered, language, publication status) used as criteria for eligibility, giving rationale. *Clearly describe eligible treatments included in the treatment network, and note whether any have been clustered or merged into the same node (with justification).* | 4 |
| Information sources | 7 | Describe all information sources (e.g., databases with dates of coverage, contact with study authors to identify additional studies) in the search and date last searched. | 4 |
| Search | 8 | Present a full electronic search strategy for at least one database, including any limits used, such that it could be repeated. | Supplementary File 1 |
| Study selection | 9 | State the process for selecting studies (i.e., screening, eligibility, included in the systematic review, and, if applicable, included in the meta-analysis). | 4 |
| Data collection process | 10 | Describe the method of data extraction from reports (e.g., piloted forms, independently, in duplicate) and any processes for obtaining and confirming data from investigators. | 4-5 |
| Data items | 11 | List and define all variables for which data were sought (e.g., PICOS, funding sources) and any assumptions and simplifications made. | 6 |
| **Geometry of the network** | **S1** | Describe methods used to explore the geometry of the treatment network under study and potential biases related to it. This should include how the evidence base has been graphically summarized for presentation, and what characteristics were compiled and used to describe the evidence base to readers. | Figure 2 and Figure 3 |
| Risk of bias within individual studies | 12 | Describe methods used for assessing the risk of bias in individual studies (including specification of whether this was done at the study or outcome level), and how this information is to be used in any data synthesis. | 6 |
| Summary measures | 13 | State the principal summary measures (e.g., risk ratio, difference in means). *Also describe the use of additional summary measures assessed, such as treatment rankings and surface under the cumulative ranking curve (SUCRA) values, as well as modified approaches used to present summary findings from meta-analyses.* | 7~8 |
| Planned methods of analysis | 14 | Describe the methods of handling data and combining results of studies for each network meta-analysis. This should include, but not be limited to:   - *Handling of multi-arm trials;* - *Selection of prior distributions in Bayesian analyses; and* - *Assessment of model fit.* | Supplementary File 6 |
| **Assessment of Inconsistency** | **S2** | Describe the statistical methods used to evaluate the agreement of direct and indirect evidence in the treatment network(s) studied. Describe efforts taken to address its presence when found. | Supplementary File 5 |
| Risk of bias across studies | 15 | Specify any assessment of risk of bias that may affect the cumulative evidence (e.g., publication bias, selective reporting within studies). | Supplementary File 7 |
| Additional analyses | 16 | Describe methods of additional analyses if done, indicating which were pre-specified. This may include, but not be limited to, the following:   - *Alternative formulations of the treatment network;* - *Use of alternative prior distributions for Bayesian analyses (if applicable).* | NA |
| **RESULTS†** |  |  |  |
| Study selection | 17 | Give numbers of studies screened, assessed for eligibility, and included in the review, with reasons for exclusions at each stage, ideally with a flow diagram. | 7~8, Figure 1 |
| **Presentation of network structure** | **A4** | Provide a network graph of the included studies to enable visualization of the geometry of the treatment network. | Figures 3 and 4 |
| **Summary of network geometry** | **A5** | Provide a brief overview of the characteristics of the treatment network. This may include commentary on the abundance of trials and randomized patients for the different interventions and pairwise comparisons in the network, gaps of evidence in the treatment network, and potential biases reflected by the network structure. | NA |
| Study characteristics | 18 | For each study, present characteristics for which data were extracted (e.g., study size, PICOS, follow-up period) and provide the citations. | Supplementary File 3 |
| Risk of bias within studies | 19 | Present data on the risk of bias of each study and, if available, any outcome level assessment. | NA |
| Results of individual studies | 20 | For all outcomes considered (benefits or harms), present, for each study: 1) simple summary data for each intervention group, and 2) effect estimates and confidence intervals. *Modified approaches may be needed to deal with information from larger networks.* | 7~8 |
| Synthesis of results | 21 | Present results of each meta-analysis done, including confidence/credible intervals. *In larger networks, authors may focus on comparisons versus a particular comparator (e.g. placebo or standard care), with full findings presented in a Supplementary. League tables and forest plots may be considered to summarize pairwise comparisons.* If additional summary measures were explored (such as treatment rankings), these should also be presented. | NA |
| **Exploration for inconsistency** | **S5** | Describe results from investigations of inconsistency. This may include such information as measures of model fit to compare consistency and inconsistency models, *P* values from statistical tests, or a summary of inconsistency estimates from different parts of the treatment network. | Supplementary File 4 |
| Risk of bias across studies | 22 | Present results of any assessment of the risk of bias across studies for the evidence base being studied. | 7, Supplementary File 7 |
| Results of additional analyses | 23 | Give results of additional analyses, if done (e.g., sensitivity or subgroup analyses, meta-regression analyses*, alternative network geometries studied, choice of prior distributions for Bayesian analyses,* and so forth). | NA |
| **DISCUSSION** |  |  |  |
| Summary of evidence | 24 | Summarize the main findings, including the strength of evidence for each main outcome; consider their relevance to key groups (e.g., healthcare providers, users, and policy-makers). | 8-10 |
| Limitations | 25 | Discuss limitations at the study and outcome level (e.g., risk of bias), and at the review level (e.g., incomplete retrieval of identified research, reporting bias). *Comment on the validity of the assumptions, such as transitivity and consistency. Comment on any concerns regarding network geometry (e.g., avoidance of certain comparisons).* | 11 |
| Conclusions | 26 | Provide a general interpretation of the results in the context of other evidence, and implications for future research. | 12 |
| **FUNDING** |  |  |  |
| Funding | 27 | Describe sources of funding for the systematic review and other support (e.g., supply of data); role of funders for the systematic review. This should also include information regarding whether funding has been received from manufacturers of treatments in the network and/or whether some of the authors are content experts with professional conflicts of interest that could affect use of treatments in the network. | NA |

PICOS = population, intervention, comparators, outcomes, study design.
